# Oligomerization is required for channel formation of MctB and for copper resistance of *Mycobacterium tuberculosis*

**DOI:** 10.1016/j.jbc.2025.111037

**Published:** 2025-12-09

**Authors:** Axel Siroy, Demeng Sun, Avraneel Paul, Jennifer L. Rowland, Lisa M. Jones, Peter Prevelige, Changlin Tian, Michael Niederweis

**Affiliations:** 1 Department of Microbiology, University of Alabama at Birmingham, Birmingham, Alabama, USA; 2 Division of Life Sciences and Medicine, University of Science and Technology of China, Hefei, Anhui, P. R. China

**Keywords:** metal homeostasis, mycobacteria, outer membrane, copper resistance, tuberculosis, virulence, structure, pore protein

## Abstract

The mammalian immune system kills bacterial pathogens including *Mycobacterium tuberculosis* by increasing copper uptake into the phagosome of infected macrophages. Rv1698 was previously identified as a membrane-spanning channel protein. The *rv1698* deletion mutant of *M*. *tuberculosis* accumulated 100-fold more copper and lungs of infected guinea pigs had a 1000-fold reduced bacterial burden compared to the WT strain. Thus, Rv1698 is an important virulence factor and was named mycobacterial copper transport protein B (MctB). However, the mechanism by which MctB confers copper resistance is unknown. Here, we solved the crystal structure of MctB which revealed a ∼7 nm long helix followed by a large globular Rossmann-like domain. Subsequent experiments showed that the N-terminal hydrophobic helix is essential for MctB export into the periplasm, for its membrane association and for its function in copper resistance. Analytical size-exclusion chromatography and gel electrophoresis showed that monomeric water-soluble MctB is in equilibrium with oligomers of molecular masses of up to ∼400 kDa. Self-assembly of MctB is induced by detergents. Importantly, only oligomeric MctB inserts into membranes in lipid bilayer experiments and forms open membrane-spanning pores capable of translocating ions. Oligomeric MctB complexes were visualized by electron microscopy. Deciphering the atomic structure of oligomeric MctB will be instrumental in understanding the molecular mechanism by which MctB contributes to copper resistance in mycobacteria.

In 2023, tuberculosis was again the world’s leading cause of death from a single infectious agent with an estimated 10.8 million infected people and 1.25 million deaths. The current treatment regimen for drug-susceptible tuberculosis requires taking four drugs for 6 to 9 months. Multidrug resistant strains of *Mycobacterium tuberculosis* cause ∼3.7% of new active tuberculosis cases and are even more challenging and expensive to treat (https://www.who.int/teams/global-tuberculosis-programme/tb-reports/global-tuberculosis-report-2023, (1), (2)). These facts highlight the necessity for new drug regimens and alternative therapeutic choices. In addition to efforts to develop new tuberculosis drugs, host-directed therapies have emerged as a promising option to enhance tuberculosis treatment regimens (3). This approach aims to treat disease by modulating the host immune system, instead of directly targeting pathogens (3). Examples of such approaches include the use of nicotinamide or derivatives to counteract the devastating effect of the tuberculosis toxin which cleaves NAD^+^, an essential cellular cofactor (4), (5) or strategies to enhance apoptosis (3) or phagosome-lysosome activity as demonstrated using the repurposed anticancer drug tamoxifen (6).

We have previously shown that the mammalian immune system utilizes copper ions to kill bacterial pathogens including *M*. *tuberculosis* (7). Upon phagocytosis of bacteria, macrophages increase copper uptake through increased expression of copper transporters CTR1 in the cytoplasmic membrane and ATP7A in the Golgi network (8). ATP7A is then relocalized to the phagosomal membrane, where it pumps Cu ions into the phagosome. Activated macrophages harboring replicating *M*. *tuberculosis*, specifically accumulate copper when infected with *M*. *tuberculosis* (9), in contrast to iron, which is actively removed from the macrophage phagosome (10). Furthermore, we have shown that copper accumulates within granulomas of guinea pigs infected with *M*. *tuberculosis* (7). Thus, during infection in *M*. *tuberculosis*, copper accumulates not only at a cellular level, but at an organ level as well.

Not surprisingly, copper resistance mechanisms have emerged as critical components of virulence of *M*. *tuberculosis* (11). The outer membrane is the primary permeability barrier of mycobacteria and requires proteins to enable uptake of small molecules (12). The outer membrane porin MspA from *Mycobacterium smegmatis* drastically increases the susceptibility of *M*. *tuberculosis* to copper ions (13), establishing that the outer membrane is indeed a major resistance mechanism against copper in *M*. *tuberculosis*. This observation suggests that copper ions diffuse across the outer membrane through water-filled membrane-spanning channels. In addition, the efflux pump CtpV (14), the copper-binding metallothionein MymT (15) and the multicopper oxidase MmcO (16) are required for copper resistance by *M*. *tuberculosis*. Eliminating one component of this multilayered defense against high copper concentrations has little to no impact on the bacterial virulence. By contrast, repression of the RicR regulon, which includes several genes involved in copper resistance, sensitizes *M*. *tuberculosis* to copper, and reduces its virulence in mice (17).

In our search for outer membrane proteins of *M*. *tuberculosis*, we have identified Rv1698 as a membrane-spanning channel protein (18). The *rv1698* deletion mutant accumulated 100-fold more copper than the WT strain, increasing its copper sensitivity and reducing the bacterial burden in the lungs of infected guinea pigs by 1000-fold and, thereby, establishing Rv1698 is an important virulence factor of *M*. *tuberculosis* (7). Based on these phenotypes, we proposed a molecular model where Rv1698 acts as the outer membrane component of the copper efflux system of *M*. *tuberculosis* (mycobacterial copper transport protein B [MctB]), similar to the tripartite copper efflux systems in Gram-negative bacteria consisting of an inner membrane efflux pump and an outer membrane channel protein (19, 20, 21). However, direct evidence for the role of MctB in copper export is still missing.

To shed light on the function of MctB, we set out to solve the structure of MctB and examine its role in copper transport on a molecular level. Surprisingly, MctB was produced in *Escherichia coli* as a water-soluble protein. The crystal structure of the MctB monomer did not reveal a membrane-compatible domain. However, detergents induce formation of MctB oligomers, which form membrane-spanning channels and are essential for copper resistance of *M*. *tuberculosis*. In this study, we show that the N-terminus of MctB is not a classical signal peptide, but is required for export, membrane association and the function of MctB in copper resistance of *M*. *tuberculosis*.

## Results

### Crystal structure of the MctB monomer

**Figure 1:**
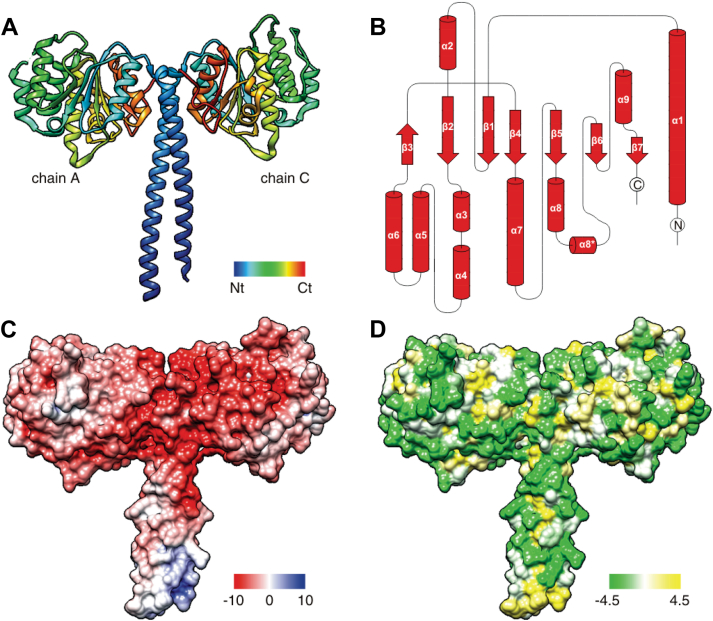
**Structure of the *M*. *tuberculosis* MctB in the presence of the detergents**. *A*, overall structure of _ΔN26_MctB_tb_ purified in the presence of C_12_E_8_. The chains A and C are depicted using a rainbow gradient from *blue* (N-terminus) to *red* (C-terminus). *B*, topology of _ΔN26_MctB_tb_ generated by PDBsum (71) and drawn with TopDraw (72). *C*, electrostatic surface of the dimer. The electrostatic surface was calculated using the UCSF Chimera Coulombic surface tool and is displayed using a color gradient from *dark red* (−10 kcal/mol) to *dark blue* (10 kcal/mol). *D*, hydrophobicity of the surface of the dimer. The hydrophobic surface was calculated using the UCSF Chimera program according to Kyte and Doolittle Hydropathy Index (73) and is displayed using a color gradient from *green* (−4.5, most hydrophilic) to *yellow* (+4.5, most hydrophobic). MctB, mycobacterial copper transport protein B.

To develop a molecular model of how MctB (Rv1698) contributes to the copper resistance of *M*. *tuberculosis*, we sought to obtain the atomic structure of MctB. Since bioinformatic analysis of the MctB N-terminus showed features of a classical Sec signal peptide, namely a positively charged amino terminus, a hydrophobic α-helix, and a predicted signal peptidase cleavage site (22), the N-terminus was replaced by a hexa-histidine tag. This recombinant MctB protein (_His6-ΔN26_MctB_tb_) was produced in *E*. *coli* and chromatographically purified to apparent homogeneity. Screening of crystallization conditions and the crystallization parameters were described previously (23). The crystal structure of _His6-ΔN26_MctB_tb_ was determined and refined to a resolution of 2.30 Å, with crystallographic parameters summarized in Table 1. The structure revealed an MctB dimer (Fig. 1*A*, chain A and chain C). The polypeptide chain was continuously traced in chain C, while several residues in chain A were not identified due to weak electron density, including the N-terminal hexa-histidine tag and the residues _124_Lys and _125_Phe. The structures of the two monomers are almost identical, with a pairwise RMSD value of 0.59 Å over the Cα atoms of 288 amino acids in chain C (Fig. S1, *C* and *D*). The overall structure of the MctB monomer comprises a C-terminal globular domain and a ∼70 Å long α helix at the N-terminus similar to a coiled-coil helix (Fig. 1*A*). At the C-terminal end of this helix, residues _71_QVG_73_ (residues 54–56 in the structural model) form a kink, followed by a loop of seven residues connecting the helix with the globular domain. This globular domain adopts a modified Rossmann fold (S85-A307) composed of seven β-strands and nine α-helices (Fig. 1*B*). A central, twisted seven-strand β-sheet is flanked by three α-helices (α1, α2, and α9) on one side and seven (α3–α8) on the other, forming an α-β-α sandwich. The β-strands are all parallel except for the β3 strand.

**Table 1:** Data collection and refinement statistics for _ΔN26_MctB_Mtb_ crystals

Dataset	Se-Met derivative			With C_12_E_8_	No detergent
Data collection					
Wavelength (Å)	0.97916 (peak)	0.97931 (edge)	0.98179 (remote)	0.97947	0.99985
Space group	*P*422	*P*422	*P*422	*P*422	*P*422
Unit cell parameters (Å, °)	a = b = 122.0, c = 87.1	a = b = 122.1, c = 87.2	a = b = 122.2, c = 87.4	a = b = 122.0, c = 88.9	a = b = 121.8, c = 88.5
	α = β = γ = 90	α = β = γ = 90	α = β = γ = 90	α = β = γ = 90	α = β = γ = 90
Resolution (Å)	20.0–2.80 (2.85–2.80)	20.0–3.00 (3.05–3.00)	20.0–3.00 (3.05–3.00)	50.0–2.30 (2.34–2.30)	50–3.25 (3.31–3.25)
Number of unique reflections				30,356	10,887
Completeness (%)	97.6 (83.3)	99.9 (99.1)	100.0 (99.2)	93.9 (56.5)	99.2 (93.4)
Redundancy	23.9 (12.9)	26.5 (20.8)	25.9 (19.7)	15.0 (9.2)	6.1 (5.1)
Average I/σ	27.7 (1.9)	36.4 (4.2)	36.8 (3.6)	64.4 (4.2)	19.1 (2.5)
R_merge_^a^ (%)	15.7 (61.1)	14.6 (63.8)	14.3 (68.4)	7.9 (42.3)	9.5 (53.5)
Refinement					
Resolution (Å)				50.0–2.30	50.0–3.25
R_work_^b^ (No. of reflections)				21.2% (27,049)	23.2% (10,306)
R_free_ (No. of reflections)				25.3% (1455)	29.7% (524)
RMSD bond lengths (Å)				0.0073	0.0079
RMSD bond angles (°)				1.2864	1.2992
Number of nonhydrogen atoms					
Protein				4130	3841
Water				99	None
Other				63	None
Average B-factors (Å^2^)				63.5	57.6
Ramachandran plot					
Most favored regions				93.1%	90.2%
Additional allowed regions				6.9%	9.8%

MctB, mycobacterial copper transport protein B.

a *R*_merg_ = |I_i_-<I>|/|I_i_|, where I_i_ is the intensity of the i^th^ measurement and <I>is the mean intensity for that reflection.

b *R*_*work*_*equals; |F*_*P*_*-F*_*P(calc)*_*|/F*_*P*_.

The electrostatic potential surface of the _His6-ΔN26_MctB_tb_ dimer is shown in Figure 1*C*. The surface of the protein dimer exhibits many negative charges, most prominently on the surface of the globular domain. The _His6-ΔN26_MctB_tb_ dimer does not present any contiguous hydrophobic stretches that could form a hydrophobic membrane anchor (Fig. 1*D*). By contrast, the protein surface is dominated by hydrophilic amino acids, except for a hydrophobic patch at the shortened N-terminus of _His6-ΔN26_MctB_tb_ (Fig. 1*D*). The dimer interface comprised of a surface area of 1925 Å^2^ with mainly hydrophobic interactions, especially along the N-terminal long coiled-coil–like region (Fig. S1).

Predictive structural models of MctB (Rv1698) were generated with AlphaFold3 (24). We produced four distinct models: full-length MctB and MctB lacking the N-terminal hydrophobic helix (_ΔN26_MctB), each as monomers and dimers (Fig. S2). We compared those models with the monomeric chain C of _His6-ΔN26_MctB_tb_ crystallized with C_12_E_8_, as the entire polypeptide chain was accounted for (Fig. S2*A*). The predictive models are in good agreement with the crystal structure as shown by the presence of an N-terminal coiled-coil helix separated from the C-terminal Rossmann domain by a hinge centered around the valine in position 72. While the C-terminal globular domain is particularly well predicted (Fig. S2*B*, <1 Å RMSD), significant deviations are observed in the peripheral loops of the C-terminal domain, its peripheral helix α4, and the coiled-coil helix towards the N-terminus. Remarkably, the deviations of the coiled-coil helix do not result from a modification of the hinge angle, but from a difference in the bending of the coiled-coil helix itself. The better global fit of the dimer models is due to the better conservation of the coiled-coil domain, indicating that interactions between MctB monomers induce changes in the coiled-coil helix. In full-length MctB, the helix forming the coiled-coil domain is ∼10 nm long compared to ∼7 nm for truncated _His6-ΔN26_MctB_tb_ (Fig. S2, *C* and *D*). Overall, these comparisons indicate plasticity in the spatial arrangement of the peripheral regions of McB extending from the core Rossmann domain.

### The N-terminus of MctB is not cleaved

**Figure 2:**
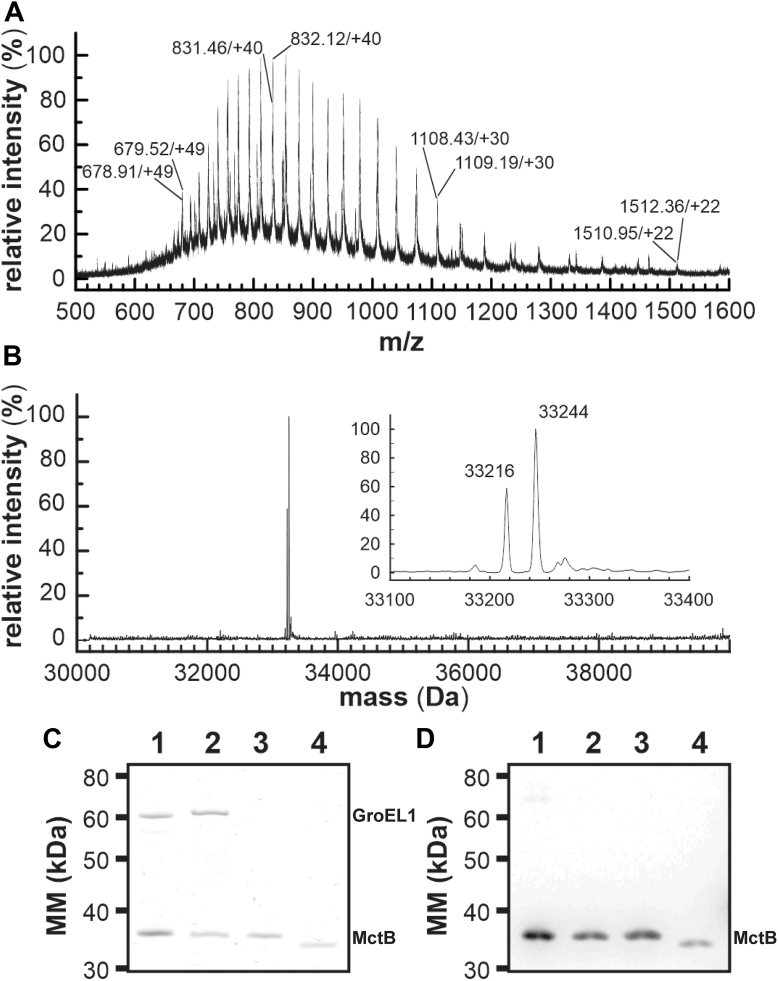
**The N-terminus of *M*. *tuberculosis* MctB is not cleaved**. *A*, electrospray ionization mass spectrum of MctB_Mtb_ purified from *M*. *bovis* BCG. The charge distribution centred around *m/z* 900 originates from 2 overlapping series of peaks corresponding to products of 33,215.97 ± 1.00 and 33,243.60 ± 1.00 Da *m/z* ratio and charge state are indicated above certain peaks. *B*, deconvolution of MctB_tb_ ESI-ToF spectrum performed using the MaxEnt program (in MassLynx, Waters) over the range 30,000 to 40,000 Da. The box highlights the region surrounding the major peak of 33,244 Da. *C* and *D*, gel electrophoretic analysis of full-length MctB proteins purified from *M*. *bovis* BCG (lanes 1) and *M*. *smegmatis* (lanes 2) containing the plasmid pML911. The ∼60 kDa band is GroEL1 which copurifies with MctB from mycobacteria cell extracts after Ni(II) affinity chromatography due its histidine-rich C-terminus (27). Recombinant full-length MctB (*lanes 3*) and truncated MctB (*lanes 4*) were produced in *E*. *coli* BL21 (DE3) Rosetta using the plasmids pML1020 and pML1021, respectively. The proteins (50 ng/lane) were separated on a 10% SDS-polyacrylamide gel and stained with Coomassie Blue G250 (*C*) and detected using an MctB-specific polyclonal antiserum after electroblotting onto a PVDF membrane (10 ng/lane) (*D*). MctB, mycobacterial copper transport protein B; ESI, electrospray ionization; BCG, Bacillus Calmette-Guérin; PVDF, polyvinylidene fluoride.

The fact that the structure of truncated MctB does not show any contiguous hydrophobic surface required for membrane insertion (Fig. 1*D*), leaves only the N-terminal hydrophobic helix, which is part of the predicted signal peptide, as a potential membrane anchor to account for the repeatedly observed membrane association of MctB (16), (18), (25). To examine whether the N-terminus of MctB is indeed cleaved as predicted, the full-length MctB_His6_ (Rv1698) protein was purified from *Mycobacterium bovis* Bacillus Calmette-Guérin (BCG). Peptide mass fingerprinting using MALDI-ToF and fourier transform ion cyclotron resonance yielded 40 peptides, which covered the complete MctB sequence except for the N-terminus (Fig. S3, Table S4), probably because hydrophobic peptides are more difficult to recover from reversed-phase chromatography columns (26). Likewise, Edman degradation did not result in any signal, indicating that the protein may be chemically blocked (not shown). In an alternative approach, we determined the mass of the full-length MctB protein by electrospray ionization mass spectrometry (ESI-MS). ESI-ToF analysis of purified MctB_His6_ produced two overlapping envelopes of masses with a charge distribution of +22 (*m/z* ∼1512) to +49 (*m/z* ∼679) (Fig. 2*A*). Deconvolution of the spectrum resulted in two peaks corresponding to molecular masses of 33,216 ± 1.0 Da and 33,244 ± 1.0 Da, respectively (Fig. 2*B*). None of the experimental values matched the calculated molecular mass for a processed MctB_His6_ protein lacking its predicted signal peptide (isotopically averaged theoretical molecular mass of 30,372.70 Da). However, the experimental molecular mass match that of unprocessed MctB_His6_. The peak of lower intensity (33,216 Da) is close to the theoretical molecular mass of the full-length MctB polypeptide (33,214.25 Da), while the peak of higher intensity (33,244 Da) indicates the presence of a formyl-methionine (_f_Met) at the N-terminus of the protein (predicted molecular mass of 33,242.25 Da). The presence of an N-terminal formyl-methionine would be consistent with the failure to chemically sequence the protein by Edman degradation. These results indicate that the N-terminus of MctB is not cleaved in *M*. *bovis* BCG.

In an alternative approach, we compared the electrophoretic mobility of MctB proteins purified from mycobacteria to those of purified recombinant full-length MctB and truncated MctB proteins lacking the N-terminal hydrophobic helix (Δ1-28). We produced the recombinant full-length MctB_His6_ and truncated _ΔN28_MctB_His6_ proteins with C-terminal hexa-histidine tags in *E*. *coli*. Both proteins were purified by Ni(II) affinity chromatography. The electrophoretic mobilities of the MctB_His6_ proteins purified from *M*. *bovis* BCG and from *M*. *smegmatis* using the plasmid pML911 containing the full-length *mctB* gene (Table S2) were identical with that of the full-length MctB protein produced in *E*. *coli* (Fig. 2*C*). By contrast, the recombinant truncated _ΔN2___8__MctB_tb_ protein lacking the predicted N-terminal signal exhibited a greater electrophoretic mobility. The identity of the purified MctB proteins was confirmed by Western blots (Fig. 2*D*). The ∼60 kDa band visible in the Coomassie-stained gel of purified MctB (Fig. 2*C*) is the mycobacterial GroEL1 chaperone, which copurified with MctB during nickel-affinity chromatography in mycobacteria because of its histidine-rich C-terminus (27). These results are consistent with the MS results demonstrating that the N-terminus of MctB is not cleaved in mycobacteria and, therefore, does not constitute a classical Sec signal sequence, which targets proteins for export across the cytoplasmic membrane (28).

### The N-terminus is required for function of MctB in copper resistance in mycobacteria

**Figure 3:**
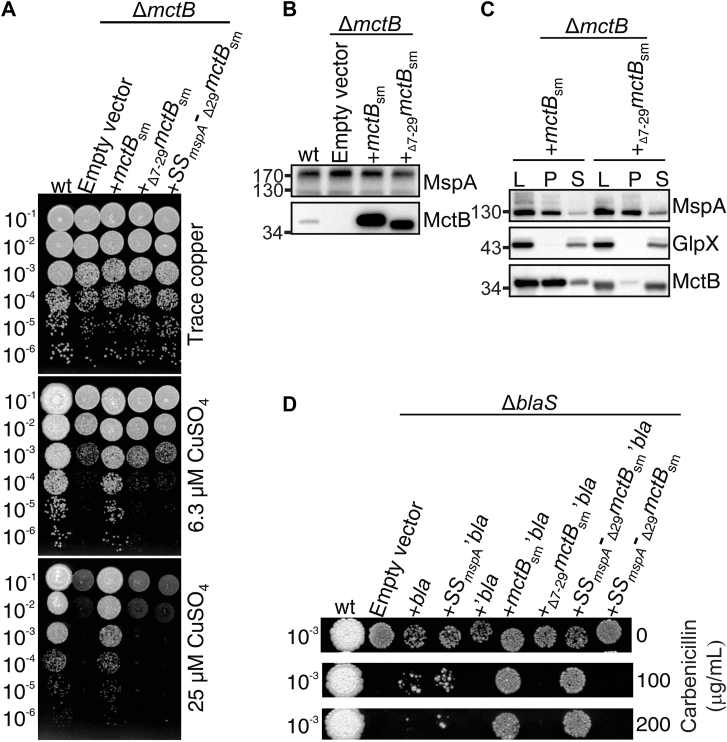
**Role of the N-terminus in the function and subcellular localization of MctB**. *A*, drop assay on agar plates containing increasing amounts of copper to determine the role of the N-terminus of MctB in copper resistance using the *M*. *smegmatis* Δ*mctB* (ML77) strain. Wt: *M*. *smegmatis* WT strain SMR5 (reference). Δ*mctB* + empty vector (pMS2; negative control); +*mctB*_sm_: pMS2 carrying *msmg_3747* (positive control); + _Δ7-29_*mctB*_sm_: pMS2 carrying *msmg_3747* encoding MctB lacking its N-terminal hydrophobic α-helix; +SS_Δ29_*mctB*_sm_: pMS2 encoding MctB_sm_ with its N-terminus replaced for the signal peptide of MspA (33), the major porin of *M*. *smegmatis* (63). *B*, comparison of protein levels of full-length MctB and _Δ7-29_MctB_sm_ in *M*. *smegmatis* ML77 compared to WT protein levels (SMR5) by Western blot analysis using the mAb 5D1.23. MspA, was used as a protein loading control. *C*, Western blot of subcellular fractions of cell lysates (L) of *M*. *smegmatis* ML77 (Δ*mctB*) expressing full-length MctB_sm_ or _Δ7-29_MctB_sm_. Soluble proteins (S) were recovered in the supernatant, while the cell envelope proteins were recovered in the membrane pellet (P). GlpX (Rv1099, Fructose 1,6-bisphosphatase) and MspA were used as cytosolic and membrane loading controls, respectively. *D*, drop assay on agar plates containing increasing amounts of carbenicillin using *M*. *smegmatis ΔblaS1* complemented with the *E*. *coli* β-lactamase TEM-1 (bla) or different fusion proteins with leaderless β-lactamase (‘bla). The full-length Western blots of Figure 3, *B* and *C* with molecular mass markers are shown in Figure S5. MctB, mycobacterial copper transport protein B.

To examine the role of the N-terminal hydrophobic helix in MctB function, we expressed full-length *mctB*_*sm*_ and *mctB*_*sm*_ lacking the predicted N-terminal hydrophobic helix (_Δ7-29_MctB_sm_) in *M*. *smegmatis*. Growth of the copper-sensitive mutant *M*. *smegmatis* ML77 (Δ*mctB*) on agar plates containing the standard concentration of 6.3 μM Cu(II) (Middlebrook 7H10 medium) was strongly reduced compared to plates with trace amounts of copper, consistent with previous results (7). Full-length MctB_sm_ restored growth of *M*. *smegmatis* ML77 to WT levels in contrast to MctB lacking the N-terminal hydrophobic helix (Fig. 3*A*). Western blot analysis of cell lysates of these strains showed that full-length and truncated protein were (over)produced at similar levels in *M*. *smegmatis* demonstrating that loss of the N-terminal hydrophobic helix did not destabilize the truncated protein or result in its degradation (Fig. 3*B*). Similar results were obtained for the MctB protein of *M*. *tuberculosis*, indicating that the MctB proteins from *M*. *smegmatis* and *M*. *tuberculosis* have the same function in copper resistance (Fig. S4). We conclude that the N-terminal hydrophobic helix is essential for the function of MctB. We therefore used the *M*. *smegmatis* strains and the *mctB*_*sm*_ expression vectors in most of the subsequent experiments.

### The N-terminal hydrophobic helix is required for membrane association of MctB in mycobacteria

To examine whether the hydrophobic N-terminal helix is required for the membrane association of MctB (16), (25), we performed subcellular fractionation experiments with *M*. *smegmatis* cells producing full-length MctB or truncated _Δ7-29_MctB. Consistent with previous results (16), (25), the majority of full-length MctB was found in the membrane fraction. However, MctB protein lacking the N-terminal helix was almost exclusively detected in the water-soluble fraction (Fig. 3*C*) demonstrating that the N-terminal helix is required for membrane association of MctB.

### The N-terminal hydrophobic helix is required for export of MctB in mycobacteria

To investigate whether MctB is indeed exported by mycobacteria, we used a β-lactamase complementation assay. *M*. *smegmatis* and *M*. *tuberculosis* are naturally resistant to β*-*lactam antibiotics primarily due to the periplasmic β*-*lactamases BlaS and BlaC, respectively (29). The *M*. *smegmatis* strain PM759 (Δ*blaS1*) does not express the endogenous β-lactamase BlaS (29), rendering it sensitive to β-lactams. Lactam resistance in this strain is fully restored by the *E*. *coli* β-lactamase TEM-1. BlaTEM-1 from *E*. *coli* is active when it is exported to the periplasm regardless whether it is translocated across the inner membrane in an unfolded form or in folded form (30), (31), in contrast to the mycobacterial β-lactamases, which require export in a folded form by the Tat system (32). Hence, we used BlaTEM-1 fusions to examine MctB translocation across the inner membrane of *M*. *smegmatis*. While all strains grow on agar plates without carbenicillin, the *M*. *smegmatis ΔblaS* mutant PM759 does not grow in the presence of carbenicillin (Fig. 3*D*). WT BlaTEM-1 with its native signal peptide restored growth of the *ΔblaS* mutant at 100 μg/ml carbenicillin, while the leaderless BlaTEM-1 (‘BlaTEM-1) only enabled growth in the presence of carbenicillin when fused to the Sec signal peptide which enables export of MspA in *M*. *smegmatis* (33) (Fig. 3*D*), confirming the compatibility of BlaTEM-1 with the Sec system (30). Growth of the *M*. *smegmatis ΔblaS* mutant in the presence of carbenicillin was also restored to WT level by the fusion of full-length MctB to leaderless ‘BlaTEM-1. In contrast, fusion of ‘BlaTEM-1 to the N terminally truncated MctB (_Δ7-29_MctB_sm_-‘BlaTEM-1) did not restore the phenotype (Fig. 3*D*). In conclusion, these experiments demonstrate that MctB is indeed exported in *M*. *smegmatis* and that its N-terminal hydrophobic helix is required for this process.

### The N-terminus has functions in addition to MctB export across the inner membrane

Next, we investigated whether the N-terminal hydrophobic helix is required only for export MctB or whether it has an additional functional role. To this end, the N-terminus of MctB was replaced with the Sec signal sequence of MspA (33). The fusion protein SS_mspA_-_Δ7-29_MctB_sm_ does not complement the copper-sensitive phenotype of Δ*mctB* (Fig. 3*A*), while the fusion protein SS_mspA_-_Δ7-29_MctB_sm_-’BlaTEM1 restores carbenicillin resistance in the *M*. *smegmatis ΔblaS* mutant PM759, demonstrating that SS_MspA_-_Δ7-29_MctB_sm_ is exported but does not provide copper resistance to *M*. *smegmatis* (Fig. 3*D*). Taken together, these results show that the Sec signal peptide mediates translocation of the MctB-lactamase fusion protein across the cytoplasmic membrane. However, export alone is not sufficient to restore the function of _Δ7-29_MctB in copper resistance indicating that MctB is either not capable of folding correctly in the periplasm when exported through the Sec translocon, or that the N-terminus is essential for MctB’s biological function, for example, as a membrane anchor.

### The proteinase K accessibility assay does not reliably detect cell surface-exposed proteins in mycobacteria

**Figure 4:**
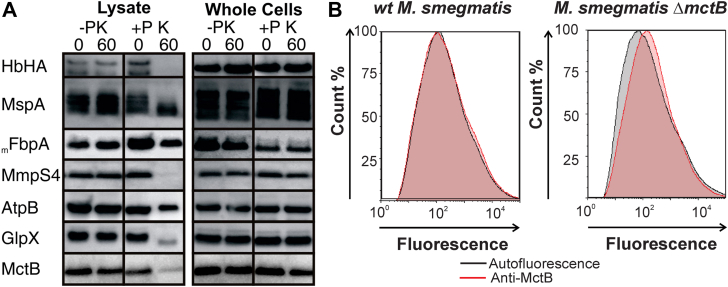
**MctB is not surface accessible in *M*. *smegmatis***. *A*, susceptibility of proteins to proteinase K in lysed and whole *M*. *smegmatis* cells. Control proteins for different subcellular localizations. Cell surface: MspA, HbHA; periplasm: MmpS4, FBPA (Ag85); inner membrane: AtpB (subunit of ATP synthase), cytosol: GlpX. *B*, the surface accessibility of MctB_HA_ was assessed by flow cytometry experiments of ∼50,000 wt *M*. *smegmatis* and *M*. *smegmatis* Δ*mctB* cells using the mAb 5D1.23. The autofluorescence of *M*. *smegmatis* is indicated in *gray*, while the green fluorescence (MctB antibody) is shown in *red*. The histogram overlay was generated by FCS Express 7. MctB, mycobacterial copper transport protein B.

The experiments described above established the essential role of the N-terminus in export and in membrane association of MctB. Previous protease accessibility assays showed degradation of MctB in whole cells after proteases treatment (18), (25), indicating that MctB might be surface-exposed and, hence, might be an outer membrane protein. However, the protease accessibility assays are known to be error-prone due to the necessity to completely inactivate the protease before cell lysis, because any remaining protease activity after cell lysis could also degrade proteins other than surface proteins. Hence, we re-examined our previous results using more stringent conditions and including more control proteins to ensure the absence of proteolytic degradation after cell lysis (not shown). Using this improved protocol, we showed that the control proteins GlpX (cytosol), AtpB (inner membrane), and MmpS4 (periplasm) were only susceptible to protease digestion in lysed cells, but not in whole cells (Fig. 4*A*). However, using this protocol even the known surface-exposed proteins MspA and HbHA (heparin-binding hemagglutinin; (34)) were not degraded by proteinase K in whole cells of *M*. *smegmatis*, although they are substrates of proteinase K as they are partial degraded in lysed cells (Fig. 4*A*). We conclude that the proteinase K accessibility assay does not reliably detect cell surface-exposed proteins in mycobacteria and, hence, is not suitable to determine the subcellular localization of MctB conclusively.

### MctB is not surface-accessible to antibodies in flow cytometry experiments

As an alternative method to detect surface-accessible proteins, we used a flow cytometry assay which is based on antibody binding to surface-exposed epitopes to detect proteins associated with the outer membrane in mycobacteria as shown previously (35, 36, 37). This method has been accepted now in the tuberculosis field (38). Quantitative analysis of ∼50,000 *M*. *smegmatis* cells by flow cytometry showed no significant binding of the monoclonal MctB antibody 5D1.23 to WT *M*. *smegmatis* and the Δ*mctB* mutant (Fig. 4*B*). Since the epitope of this antibody is located on the outside of the Rossman-like globular domain of MctB (Fig. S6) and should be easily accessible by the antibody, we conclude that this domain of MctB is not surface-exposed in *M*. *smegmatis*.

### The N-terminal helix of MctB stabilizes oligomers

**Figure 5:**
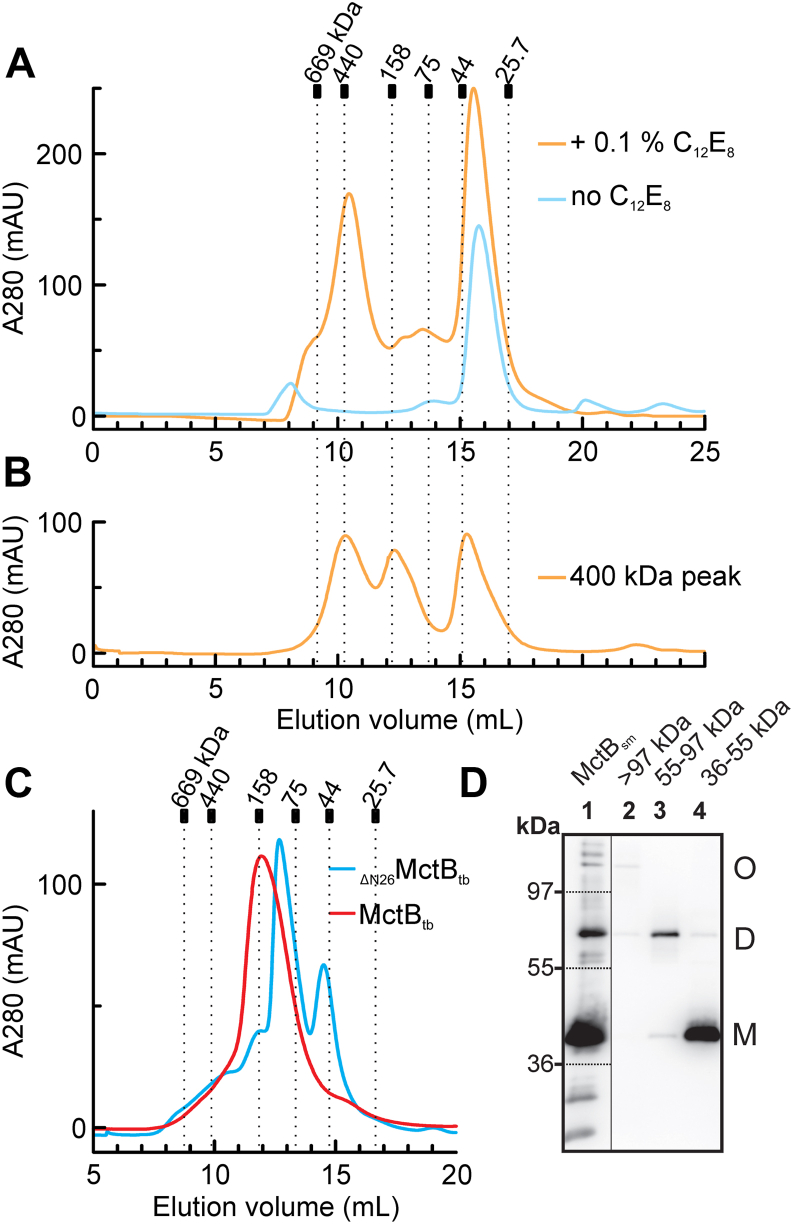
**Detergents induce oligomerization of *M*. *tuberculosis* MctB and isolation of monomeric and oligomeric MctB**. *A*, purified _ΔN26_MctB_tb_ protein (>90% pure) was analyzed by size-exclusion chromatography in the absence of (*blue line*) or in the presence of the detergent C_12_E_8_ (*orange line*). *B*, analysis of oligomeric MctB fractions (∼400 kDa peak) from run (*A*) by size-exclusion chromatography in the presence of C_12_E_8_. *C*, isolation by size-exclusion chromatography of_ΔN26_MctB_tb_ (*blue line*) or MctB_tb_ (*red line*) oligomers purified in the presence of LDAO. All chromatography was done using a Superdex200 10/300 column. The column calibration curve using molecular mass markers and the molecular masses calculated for the MctB monomer and oligomers are shown in Figure S8. *D*, *Lane 1*: purified MctB_sm_ was analyzed by gel electrophoresis using using “mild” conditions: the protein loading buffer contained less SDS (0.1%), no reducing agent and the samples were not boiled to enable detection of oligomers. MctB monomers (m), dimers (d), and oligomers (o) were excised from an SDS-polyacrylamide gel and extracted using the detergent OPOE. The fractions are indicated by *dotted lines*. The eluted proteins were separated by gel electrophoresis using “mild” conditions as described above and stained using the monoclonal MctB antibody 5D1.23 in a Western blot. *Lane 2*: proteins >100 kDa (o); lane 3: 55 to 100 kDa (d); lane 4: 36 to 55 kDa (m). The complete SDS-PAGE and Western blot analyses of the gel purification of recombinant rRv1698 from *E*. *coli* and native Ms3747 from *M*. *smegmatis* are shown in Figure S9. LDAO, lauryldimethylamine oxide; MctB, mycobacterial copper transport protein B; OPOE, n-octyl-oligo-oxyethylene.

Since the crystal structure of the MctB monomer did not reveal a membrane spanning domain except for the N-terminal hydrophobic helix, we examined whether MctB forms oligomers as most membrane channel proteins are oligomers. To this end, we replaced the N-terminus including the hydrophobic helix of MctB from *M*. *tuberculosis* with a hexa-histidine affinity tag. This protein was produced in *E*. *coli* and purified using Ni(II) affinity and size-exclusion chromatography (SEC) as previously described (23). Calibrated SEC showed that _His6-ΔN26_MctB_tb_ is mainly a monomeric protein in the absence of detergents (Fig. 5*A*). However, in the presence of the nonionic detergent octaethylene glycol monododecyl ether (C_12_E_8_), _His6-Δ26_MctB_tb_ revealed an additional oligomeric form of ∼400 kDa (Fig. 5*A*). The oligomeric fractions of this peak were pooled and subjected again to analytical SEC under the same conditions. In this experiment, MctB eluted in the three distinct peaks of ∼400, ∼150, and ∼40 kDa indicating that the ∼400 kDa MctB oligomer is in equilibrium with an intermediate and a monomeric form in the presence of detergents (Fig. 5*B*).

The lack of large hydrophobic surfaces in the initial crystal structure (Fig. 1*D*) and the presence of N-terminally truncated MctB_sm_ in the water-soluble fraction of *M*. *smegmatis* (Fig. 3*C*) suggested that it might be possible to purify _His6-Δ26_MctB_tb_ in the absence of detergent. Indeed, the protein was present in the water-soluble fraction of *E*. *coli* lysates, was purified by Ni(II) affinity chromatography and eluted mainly as a monomer (∼40 kDa) from the SEC column similar to the chromatogram shown in Figure 5*A*. The MctB monomer was crystallized, diffraction data were collected, and the structure was determined and refined to a resolution of 3.25 Å using the previous structure of detergent-purified MctB monomer for molecular replacement. The pairwise RMSD of the two structures is 0.455 Å for 516 Cα atomic pairs (Fig. S7). The identity of both structures demonstrates that the detergent C_12_E_8_ does not change the structure and/or the oligomeric state of MctB in the crystal, although C_12_E_8_ induces an equilibrium shift of MctB from the monomeric to the oligomeric state (Fig. 5*A*).

### Oligomeric MctB forms membrane-spanning channels in lipid bilayer experiments

Western blot analysis of full-length MctB_sm_ (from *M*. *smegmatis*) purified in the presence of the nonionic detergent n-octyl-poly-oxyethylene (OPOE) showed discrete bands with apparent molecular masses larger than the 37 kDa monomer (Fig. 5*D*). The additional bands correspond to SDS stable dimers at ∼70 kDa and several larger MctB oligomers with apparent molecular masses above 100 kDa. To enrich these different species, the purified MctB_sm_ protein sample (>90% MctB) was separated on a preparative SDS-polyacrylamide gel. Four different parts of the gel corresponding to molecular masses of > 97 kDa, 55 to 97 kDa, 36 to 55 kDa, and 10 to 36 kDa were cut and their protein content was eluted using an OPOE-containing buffer. These fractions were analyzed by SDS-PAGE and subsequent Western blots. Proteins in the ≥100 kDa fraction partially converted to a ∼70 kDa band corresponding to an MctB dimer. Proteins in the ∼70 kDa fraction partially converted to monomeric MctB (∼37 kDa) and monomeric MctB partially converted to the dimer (Figs. 5*D*, S9). We applied the same protocol of isolation from polyacrylamide gel to MctB_tb-His6_ expressed in *E*. *coli* and purified from inclusion bodies using Ni(II) affinity and SEC as previously described (16). MctB_tb-His6_ freshly purified in the presence of OPOE was incubated at 4 °C and 20 °C for a week before the observation of oligomer forms of similar sizes and their extraction from the gel in the same conditions (Fig. S9). Then, these samples were heated in protein loading buffer with 0.1% SDS (10-fold reduced concentration) to 37 or 100 °C and analyzed after gel electrophoresis in silver-stained gels and by Western blots. These experiments clearly show the presence of heat-stable MctB oligomers (Fig. S9, *A* and *B*). Importantly, these experiments also show defined, interconvertible oligomeric bands which are identical between native MctB purified by detergent extraction from its host *M*. *smegmatis* and the recombinant, refolded MctB protein purified from inclusion bodies from *E*. *coli* (Fig. S9), indicating that MctB has the intrinsic capacity to form stable oligomers and are not refolding artefacts of the recombinant protein. It should also be noted that MctB does not contain cysteines, ruling out the possibility that the observed MctB oligomers result from disulfide bond formation under oxidizing conditions. In conclusion, these results indicate an equilibrium between the monomeric and oligomeric forms of MctB and are consistent with the SEC results.

**Figure 6:**
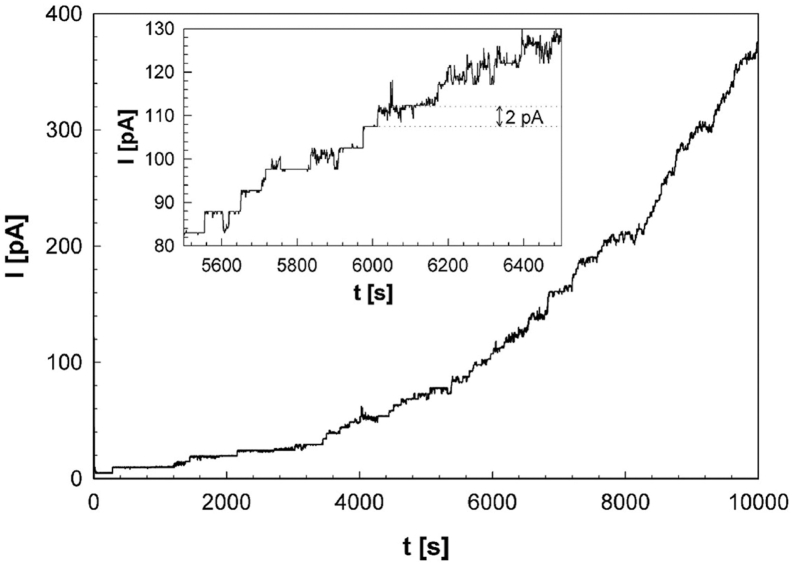
**MctB oligomers form membrane-spanning channels**. Purified MctB_sm_ as shown in Figure 5*D* (lane 1) was dialyzed and analyzed in lipid bilayer experiments. We used diphytanoyl phosphatidylcholine (DphPC) membranes bathed in 1 M KCl, 10 mM Hepes (pH 7.0) as an electrolyte, and recorded current traces at 100 mV applied potential. The gain of the amplifier was 10^9^ V/A. No current was detected when only buffer was added (not shown). A representative current trace shows the continuous insertion of open MctB channels for more than 2 h when MctB protein was added to both sides of the cuvette. The inset shows a 15-min window of the same current trace to visualize the stepwise current increase after addition of MctB. MctB, mycobacterial copper transport protein B.

The purified MctB protein before fractionation and the three fractions eluted from the polyacrylamide gel were analyzed in lipid bilayer experiments to examine their pore-forming activities. While no pores were detected using the detergent-containing buffer alone, the purified MctB protein before fractionation showed sustained membrane insertions as indicated by stepwise current increases for more than 2 h (Fig. 6). The observed insertions of ∼2 pA correspond to channels with an average conductance of 20 pS at a potential of 100 mV. No channel activity was observed with the fraction after preparative gel electrophoresis containing MctB monomers (Fig. 5*D*, lane 4) (not shown). By contrast, the MctB dimer and oligomer fractions showed pore-forming activity, albeit at lower levels than the purified MctB protein sample, probably due to the denaturing activity of the SDS-containing loading buffer and gel (not shown). These observations are consistent with a model that the MctB monomer is capable of forming stable oligomers and that oligomerization is required for formation of membrane-spanning channels.

### Negative-stain electron microscopy reveals stable MctB oligomers

To obtain structural information about the MctB oligomer, we screened detergents to stabilize the oligomeric form of MctB. MctB was overproduced in *E*. *coli* and extracted with lauryldimethylamine oxide (LDAO). Purification in the presence of LDAO shifted the equilibrium completely to oligomeric forms of MctB. Analytical SEC revealed apparent molecular masses of ∼220 kDa and ∼160 kDa for _His6_MctB_tb_ and _His6-ΔN26_MctB_tb_, respectively (Fig. 5*C*). Since MctB oligomers purified in the presence of LDAO did not give crystals, we used electron microscopy of grids stained with uranyl acetate to visualize the MctB particles (Fig. S10). The hydrophilic uranyl ions do not accumulate at hydrophobic regions of the protein but render hydrophilic surfaces opaque to the electron beam. Approximately 1000 particles of MctB protein were clustered and several class-averaged images showed oligomeric MctB (Fig. S10), demonstrating that detergents such as LDAO induce oligomerisation of MctB. This finding is consistent with the SEC results shown in Figure 5.

## Discussion

### Structure of the MctB monomer

We solved the structure of recombinant MctB from *M*. *tuberculosis* in the presence or absence of the nonionic detergent C_12_E_8_ by X-ray crystallography. The _His6-ΔN26_MctB_tb_ protein crystallized as a pair of dimers. Each monomer comprised two main domains: a long N-terminal α-helical domain followed by a C-terminal globular domain forming the bulk of the structure (covering ∼70% of the sequence). The globular domain is arranged in a modified double Rossmann fold, akin to the dinucleotide binding domains found in several cytoplasmic enzymes (39), (40). When _His6-ΔN26_MctB_tb_ was purified as a monomer without detergent and crystallized, the structure did not show any significant difference demonstrating that MctB_tb_ lacking its N-terminal hydrophobic helix is water soluble. The crystal structure of _ΔN26_MctB_tb_ from oligomeric fractions obtained in the presence of C_12_E_8_ showed the same conformation as in crystals from monomeric MctB. These results support the hypothesis that MctB exists in equilibrium between the monomer and oligomeric forms and indicate that the oligomeric structure of _ΔN26_MctB_tb_ is formed by assembly of monomers without major changes of its structure. However, the structure of the MctB monomer did not show any features compatible with the observed integration into membranes and channel formation by MctB. Specifically, the structure did not reveal a continuous hydrophobic belt or an open channel necessary to integrate into biological membranes and form pores. A major structural rearrangement from the current mixed α/β-structure to an oligomeric β-barrel pore structure would be energetically expensive and is therefore unlikely. However, such a model has been proposed for OmpA proteins from Gram-negative bacteria (41), (42), whose crystal structures show small, membrane-integrating β-barrels, but no continuous water-filled pores (43). Such a model is supported by indirect evidence that OmpA plays a role in uptake of small and water-soluble molecules such as β-lactam antibiotics (44) and by direct evidence for channel formation of OmpA and homologs in lipid bilayer experiments (45, 46, 47). Molecular dynamic simulations indicate some degree of pore expansion (48) consistent with structural flexibility revealed by NMR structure of OmpA (49). While these publications establish OmpA proteins as an example of a bacterial outer membrane protein with considerable structural flexibility, much larger structural changes would be necessary for the MctB monomer to adopt a pore-forming structure. For this reason, we favor the hypothesis that oligomerisation of MctB is required for membrane integration and channel formation.

### Pore formation of oligomeric MctB in “classical” membranes and mycobacterial outer membranes

Our experiments show that the hydrophobic N-terminus is an integral part of the mature MctB protein and is essential for its export into the periplasm, its membrane association and its function in copper resistance of *M*. *tuberculosis*. In this study, we also purified the *M*. *smegmatis* MctB homolog and showed that it forms open channels in lipid membranes similar to full-length MctB from *M*. *tuberculosis* (Rv1698) purified from both *E*. *coli* and *M*. *bovis* BCG (18). The pore activity of purified MctB in lipid bilayer experiments is also consistent with its pore activity in mycobacteria as it partially complements a porin mutant of *M*. *smegmatis* (18). Importantly, pore formation in lipid bilayers was only detected for MctB oligomers but not for the MctB monomer (Fig. 6). The formation of oligomeric MctB is supported by SEC, gel electrophoresis, and electron microscopy data. We propose that the N-terminal hydrophobic helices of MctB monomers assemble to a coiled-coil domain and form a continuous hydrophobic surface. Considering that the contiguous hydrophobic stretch of the N-terminal helix in full-length MctB is ∼3.3 nm exceeds the length of 2.5 nm required by a hydrophobic helix to span a lipid membrane (50), indicating that a putative hydrophobic coiled-coil domain of the N-termini in an MctB oligomer is sufficient to span the hydrophobic core of conventional biological membranes. Previous experiments in a mutant of *M*. *smegmatis* lacking the outer membrane porins MspA and MspC showed that glucose uptake is significantly increased upon expression of *mctB* demonstrating that the MctB channel can also traverse the outer membrane in mycobacteria (18). This transport activity of MctB in *M*. *smegmatis* is also consistent with the observation that the hydrophobic part of the membrane-spanning β-barrel of MspA is only ∼3.5 nm thick (51). Rings of aromatic amino acids, which often delineate membrane-spanning domains of proteins, indicate that membrane-spanning domain of MspA might be as short as ∼2.2 nm (52). In conclusion, these results indicate that the thickness of the hydrophobic core of mycobacterial outer membranes is significantly shorter than the overall membrane thickness of 7.5 nm (53) and that a putative hydrophobic domain of an MctB oligomer with a length of ∼3.3 nm is sufficient to span mycobacterial outer membranes. In this regard, it is important to note that the electron micrographs of negatively stained MctB do not show an open pore (Fig. S10). Thus, interactions with a membrane as in the lipid bilayer experiments (Fig. 6), with a translocation substrate and/or other proteins may be required for MctB to transition to an open pore.

### Does the pore activity of MctB explain its function in copper resistance of mycobacteria?

Previously, we postulated that MctB might be involved in copper efflux to account for the 100-fold increased accumulation of copper ions in the *mctB* deletion mutant of *M*. *tuberculosis* (7). Such a model appeared to be attractive since inner membrane copper efflux pumps such as CtpV are known in *M*. *tuberculosis* (11), (14), (54), but a corresponding outer membrane component was missing. However, in this study, we did not find direct evidence supporting the localization of MctB in the outer membrane. By contrast, we show that previous protease accessibility experiments (18) are error prone and, using more stringent conditions, we did not detect MctB on the surface *M*. *smegmatis* cells. However, in these experiments we also did not detect the outer membrane protein MspA despite it is a large extracellular domain (52). Flow cytometry of *M*. *smegmatis* cells also did not detect the epitope of the mAb, which is located on the outside of the hydrophilic Rossman-like domain of MctB (Fig. S5), and would be accessible on the cell surface if this domain were exposed. Thus, we conclude that the Rossman-like domain of MctB is not on the cell surface. On the other hand, we clearly show that MctB is exported and associated with membranes and that MctB fusion proteins are active in the periplasm. In addition, integration of proteins with open channels such as MctB (Fig. 6) into the inner membrane of bacteria is unlikely because such events would be lethal due to the rapid loss of small molecules and ions including protons. Based on these considerations and our experimental results, we propose that MctB is associated with the outer membrane in a reverse orientation with the hydrophilic Rossman-like domain facing the periplasmic side.

Another prerequisite for a direct role of MctB in copper resistance by *M*. *tuberculosis* is that the MctB pore is permeable for copper ions. While we did not measure the conductivity of the MctB channel using CuCl_2_ as an electrolyte, previous zero current potential measurements revealed a weak preference for cations (18). In this previous study, we also show that the MctB channel is permeable for lithium and sodium ions in addition to potassium ions. This is important since the radius of a lithium ion (76 pm) is similar to the radius of a copper (II) ion (73 pm). In addition, the radius of the hydrated copper (II) ion is smaller than the hydrated lithium ion since its nucleus has a higher charge. Thus, we conclude that copper (II) ions can diffuse through the MctB pore as part of a copper efflux system in *M*. *tuberculosis*. However, we do not have direct evidence for such a mechanism.

## Conclusions

The formation of oligomers solves the conundrum how the water-soluble MctB monomer is capable of assembling a membrane-spanning pore and is consistent with the biochemical, biophysical, and structural data in our previous report (18) and in this study. In this model, the N-terminal hydrophobic helix of MctB serves as a membrane anchor, with the majority of the protein located in the periplasm. This membrane anchor would increase the local MctB population and hence promote oligomerization of MctB. Deciphering the atomic structure of oligomeric full-length MctB by cryo-EM will be instrumental in understanding the molecular mechanism by which MctB contributes to copper resistance in mycobacteria.

## Experimental procedures

### Strains and plasmids

All bacterial strains and plasmids used in this study are listed in Table S1. Mycobacterial strains were grown at 37 °C in Middlebrook 7H9 liquid medium (Difco Laboratories) supplemented with 0.2% glycerol, 0.02% tyloxapol (Sigma-Aldrich) or on Middlebrook 7H10 agar (Difco Laboratories) supplemented with 0.2% glycerol unless indicated otherwise. *E*. *coli* DH5α was used for all cloning procedures. 7H9 or 7H10 medium lacking copper (7H9 trace copper, 7H9tc) was made as previously described (7), and copper was added in the form of CuSO_4_ when required. *E*. *coli* BL21(DE3) and C43(DE3) were used for the expression of recombinant proteins. The *E*. *coli* strains were routinely grown in Lysogenic Broth medium at 37 °C unless indicated otherwise. The following antibiotics were used when required at the following concentrations: ampicillin (100 μg/ml for *E*. *coli*), kanamycin (30 μg/ml for *E*. *coli* and *M*. *smegmatis*), and hygromycin (200 μg/ml for *E*. *coli*, 50 μg/ml for *M*. *smegmatis*). Detailed cloning procedures for each plasmid are described in the Supplemental Materials.

### Bioinformatic analysis

The sequences of the genes coding for MctB in *M*. *tuberculosis* and *M*. *smegmatis* were obtained from the MycoBrowser server (http://mycobrowser.epfl.ch). The *rv1698* and *msmeg_3737* genes were named *mctB*_tb_ and *mctB*_sm_ in this work. The amino acid sequences of the encoded MctB proteins were analyzed with the SignalP and TMHMM algorithms (22), (55).

### Subcellular fractionation of *M*. *smegmatis*

The subcellular localization of proteins was determined by fractionation experiments as previously described (16), (56). Briefly, clump-free cultures of *M*. *smegmatis* were grown to mid-exponential phase in self-made Middlebrook 7H9 medium with tyloxapol and no added copper (7H9tc medium). Cells were harvested by centrifugation and washed with ice-cold PBS buffer twice to eliminate the detergent. The pellets were resuspended in ice-cold PBS and the final volumes normalized for an optical density at 600 nm (OD_600_) of 50. The cell suspensions were suspended with 1 mM EDTA pH7.6, 2 mM PMSF, 0.1 unit/ml benzonase, 1 mg/ml lysozyme, sonicated twice and left on ice for 30 min. The lysates were then cleared by centrifugation. The membrane fraction was separated from the soluble content of the clear lysate by ultracentrifugation (two steps of 165,000×*g*, 4 °C, 60 min). The resulting pellet fractions were combined, dispersed in PBS buffer by sonication, and the volumes of the pellet and supernatant fractions were normalized prior to their analysis by SDS-PAGE and Western blot.

### Copper susceptibility assay

Agar plates with self-made Middlebrook 7H10 medium supplemented with hygromycin and tyloxapol were prepared with different concentrations of CuSO_4_ (trace copper, 6.3 and 25 μM) and left to dry overnight as previously described (7). Cultures were grown overnight in liquid 7H9tc medium supplemented with hygromycin and tyloxapol and subsequently diluted to an OD_600_ of 0.1 with sterile PBS containing 0.02% tyloxapol (noted dilution 10^-1^). Successive dilutions (1:10) of the cell suspensions were performed in the same buffer, from 10^-2^ to 10^-6^. Three microliters of each dilution for each strain were dropped on the dry 7H10_tc_ containing increasing concentrations of CuSO_4_ as indicated in the figures. The plates were incubated at 37 °C for 3 days prior to taking images.

### β-Lactam susceptibility assay

Plasmids encoding the proteins to be tested were transformed into *M*. *smegmatis* strain PM759 lacking the endogenous β-lactamase gene *blaS1* (see Table S1) (29). PM759 strains were grown in 7H9 or 7H10 containing L-lysine (80 μg/ml or 40 μg/ml, respectively), as PM759 is a lysine auxotroph. 7H10 plates supplemented with hygromycin and tyloxapol were prepared with different concentrations of carbenicillin (0, 100, 200 μg/ml). Clump-free cultures were grown overnight in liquid 7H9 medium supplemented with hygromycin, tyloxapol, and 80 μg/ml L-lysine (for PM759 strains). Single cell suspensions were washed and subsequently diluted to an OD_600_ of 0.1 with sterile PBS containing 0.02% tyloxapol (noted dilution 10^-1^). Successive dilutions (1:10) of the cell suspensions were performed in the same buffer, from 10^-2^ to 10^-6^. Three microliters of each dilution for each strain were spotted on 7H10 plates containing the increasing concentrations of carbenicillin. The plates were incubated at 37 °C until colonies were visible at the lowest dilution before images were captured.

### MctB production in *E*. *coli* and purification for functional analysis

Cultures of *E*. *coli* Rosetta carrying the overexpression vectors pML1020 (*rv1698*_His6_) and pML1021 (_ΔN28_*rv1698*_His6_) were grown at 37 °C. When the culture reached an OD_600_ of 1, IPTG was added to a final concentration of 0.5 mM to induce gene expression. After 4 h of incubation, the bacteria were harvested and resuspended in 20 ml of lysis buffer (50 mM Tris–HCl pH 8, 100 mM NaCl, 0.5% Triton X-100). The cell suspension was sonicated four times for 20 s with 12 W in 30-s intervals. A total of 0.01 mg/ml DNase and 0.1 mg/ml lysozyme were added and incubated at room temperature for 20 min. The broken cells were harvested by centrifugation and resuspended in lysis buffer followed by sonication as described above. This step was repeated twice. Then, the broken cells were resuspended in washing buffer (50 mM Tris–HCl pH 8, 100 mM NaCl) and sonicated again. Because recombinant MctB formed inclusion bodies, the proteins in the pellets were dissolved with 8 M urea, separated from cell debris by centrifugation, and purified by gravity-flow chromatography using nickel-nitrilotriacetic (Ni-NTA) agarose as solid phase (Qiagen). The bound proteins were washed in three steps with five bed volumes of washing buffer containing 10 mM imidazole, 0.1% SDS, and decreasing concentrations of urea (6 M, 4 M, and 2 M, respectively). The bound proteins were recovered with three bed volumes of elution buffer containing 250 mM imidazole, 0.1% SDS, and 1 M urea. Urea and imidazole were further removed by dialysis with using 10 kDa cut-off Slide-A-Lyzer cassettes (Pierce) and a buffer containing 50 mM Tris–HCl (pH 8), 100 mM NaCl, and 0.1% SDS. Protein concentrations were determined using a Bradford assay (Bio-Rad).

### Purification of MctB for crystallography

Recombinant MctB_tb_ lacking its 26 N-terminal amino acids and harboring an N-terminal hexa-histidine tag (referred to as _His6-ΔN26_MctB_tb_) was expressed in *E*. *coli* BL21(DE3) and purified as previously described (23). Briefly, the protein was extracted with the nonionic detergent octaethylene glycol monododecyl ether (C_12_E_8_) from the cleared lysate of *E*. *coli* and purified by immobilized metal affinity chromatography using a Ni-NTA resin, followed by SEC to obtain pure fractions of MctB.

### Crystallization of MctB

Since no homologous structure was available for molecular replacement, the structure of _His6-ΔN26_MctB_tb_ was determined using the multiwavelength anomalous dispersion method (57). Seleno-methionine (Se-Met)-labeled _His6-ΔN26_MctB_tb_ was prepared following standard procedures in minimal medium M9 using *E*. *coli* strain B834 (Novagen). Procedures for purification and crystallization of the Se-Met derivative _His6-ΔN26_MctB_tb_ were identical to those of the native protein.

Initially, native _His6-ΔN26_MctB_tb_ was crystallized using the sitting-drop vapor diffusion method at 295 K. The purified protein was concentrated to 6 mg/ml in a solution of 50 mM Tris–HCl pH 8.0, 500 mM NaCl, 5% (v/v) glycerol, and 0.1% (w/v) C_12_E_8_. One microliter protein solution was mixed with an equal volume of reservoir solution, and the droplet was equilibrated against 100 µl reservoir solution. After 1 week, microcrystals appeared in a condition consisting of 0.1 M sodium acetate pH 4.6, 2 M ammonium sulfate. Following optimization of the crystallization condition, the best _His6-ΔN26_MctB_tb_ crystals for data collection were grown in 0.1 M sodium acetate pH 4.6, 1.8 M ammonium sulfate with a protein concentration of 5 mg/ml at 295 K. The crystal was harvested, soaked in a cryoprotectant solution supplemented with 20% (w/v) glycerol, and then was flash-cooled in liquid nitrogen.

X-ray diffraction data were collected on beamline BL17U at the Shanghai Synchrotron Radiation Facility. For native _His6-ΔN26_MctB_tb_, 180 images were recorded from one single crystal at 100 K. The diffraction data were processed using HKL-2000. For the Se-Met derivative of _His6-ΔN26_MctB_tb_, a multiwavelength anomalous dispersion experiment was performed using a single Se-Met–derived crystal cryo-cooled to 100 K. Diffraction data were collected at three wavelengths corresponding to the peak, inflection point, and a remote low-energy wavelength, as optimized from an X-ray fluorescence scan (Table 1). All datasets were integrated and scaled using the HKL-2000 software package. The positions of the selenium atoms were located and the initial phases were calculated using the Phenix program package (58). The substructure solution and phasing statistics were as follows: Bayes-CC = 0.45 and figure of merit = 0.118. An initial model was automatically built into the experimental electron density map using Autobuild in the Phenix program package (82). After tracing the initial model manually in the program COOT (59), the model was then refined against the native _His6-ΔN26_MctB_tb_ data using the Crystallography & NMR system (60) and REFMAC5 (61), and rebuilt interactively in COOT. The model was refined to a resolution of 2.30 Å and R/Rfree factor of 21.2%/25.3% finally (Table 1).

The _His6-ΔN26_MctB_tb_ protein purified in aqueous solution (without detergent, denoted as MctB_nodet_) crystallized under the same conditions as the protein purified in the presence of detergent. Using the determined _His6-ΔN26_MctB_tb_ structure as a searching model, the structure of MctB_nodet_ was solved by the molecular replacement method using the MOLREP program (62). The model was refined to 3.25 Å using REFMAC5 and COOT alternatively, resulting in a final model with an R-factor of 23.2% and free R-factor of 29.7% (Table 1). The statistics for the X-ray data collection and the structures refinement are listed in Table 1. The refined models have been deposited in the Protein Data Bank with PDB ID: 9M9Z (_His6-ΔN26_MctB_tb_) and 9MA1 (MctB_nodet_).

### Western blot analysis

Protein samples were analyzed by SDS-PAGE and Western blot analysis according to standard protocols. Briefly, after separation on polyacrylamide gels, the proteins were transferred on a polyvinylidene fluoride membrane. The membranes were blocked with Tris-buffered saline with Tween (TBST) containing 5% nonfat dry milk (m/v). The membranes were subsequently probed with primary antibodies in TBST plus milk in the same conditions. The following primary antibodies were used: monoclonal α-MctB_Mtb_ antibody 5D1.23 (7), polyclonal α-Msmeg_3747 antiserum RB27083 (recognizes MctB in *M*. *smegmatis* only), the polyclonal α-MspA antiserum pAK81 (63) and the polyclonal antiserum anti-GlpX (56). Then, the blots were washed three times for 5 min with TBST, incubated with goat anti-rabbit or goat anti-mouse secondary antibodies conjugated to horseradish peroxidase (Sigma) in TBST plus milk and washed again under the same conditions. Then, the blots were analyzed with an enhanced chemiluminescence Western blotting substrate (Pierce) and a LabWorks imaging system (UVP) to visualize and quantify the luminescence of the blots. The software Gimp was used to adjust the contrast of the images. Quantitative analysis was performed on raw images with the ImageJ image analysis package (Rasband, W.S., ImageJ, US National Institutes of Health, https://imagej.net/ij/). The integrated luminescence signal was measured in the analyzed lanes and the results were normalized to the values obtained for the constitutively expressed proteins MspA and/or GlpX.

### Analytical SEC

The oligomeric states of _His6-ΔN26_MctB_tb_ in the presence or absence of detergent were analyzed using SEC. A Superdex 200 10/300 column (GE Healthcare) was pre-equilibrated with the running buffer (20 mM Tris pH 8.0, 200 mM NaCl) in the presence or absence of 0.1% (w/v) C_12_E_8_. Five hundred microliters of sample were loaded on the column using an injection loop and UV absorption was monitored at 280 nm. The column was calibrated using six marker proteins (Bio-Rad) and the molecular masses of the proteins of interest were derived from the standard curve. The oligomeric state of _His6_MctB_tb_ was analyzed in the same conditions using running buffer supplemented with 0.1% (w/v) C_12_E_8_ or 0.1% (w/v) LDAO.

### Isolation of MctB_sm_ from *M*. s*megmatis*

Full-length MctB_sm_ was purified from *M*. *smegmatis* ML77 containing the plasmid pML948 carrying the *ms3747* gene under the control of the acetamidase promoter (64), (65). Briefly, the expression was induced by the addition of acetamide in the culture, the cells were harvested, lysed by sonication, the protein was extracted with the non-ionic detergent OPOE and purified by affinity chromatography on Blue-Dextran agarose followed by anion-exchange and SEC. The purified MctB_sm_ was concentrated to 1 mg/ml and stored at 4 °C.

### Peptide mass fingerprinting of MctB

Recombinant _ΔN28_MctB_His6_ was purified as described before (18). The purified proteins were run on SDS-PAGE, stained with brilliant Coomassie Blue G250 and subjected to in-gel trypsin digestion. To this end, the stained protein bands were excised from SDS-polyacrylamide gels, destained, and digested with sequencing grade trypsin (Roche) using a protein-trypsin ration of 20:1 at 37 °C overnight. The protein digestion was quenched with 1% formic acid and peptides were extracted by diffusion (66). Peptides digested in gel were analyzed by nano liquid chromatography fourier transform ion cyclotron resonance (LCFT-ICR MS, Thermo Fisher Scientific). The sample was diluted to a concentration of less than 200 ng/μl and loaded onto a prepacked C18 300 Å column using an autosampler (Eksigent). The protein was eluted using a linear gradient from 5% to 40% acetonitrile containing 0.1% formic acid for 1 h at a flow rate of 500 nl/min. Tandem mass spectrometry sequencing was used to identify the peptides.

### Preparative gel electrophoresis of MctB

For the isolation of MctB oligomers by SDS-PAGE, the following conditions were modified from standard procedures: final concentration of SDS in the loading buffer was lowered from 1% to 0.1% (m/v), reducing agents were not used (no cysteines in MctB) and the sample was not heat-denatured prior to the electrophoresis step that was performed in an ice bath.

### Electrospray mass spectroscopy of MctB

Full-length MctB_tb_ with a C-terminal hexahistidine tag was purified as described (18). The fraction eluted from the Ni-NTA column containing full-length MctB_His__6_ purified from *M*. *bovis* BCG coeluted with GroEL1. Further purification was achieved by preparative PAGE. The gels were stained using reversible negative staining with imidazole-zinc salts (67). Subsequently, the gels were rinsed two times in deionized water for 30 s and then incubated in a solution of 0.1% SDS, 0.2 M imidazole for 15 min. The solution was discarded, and the gels were incubated in 0.2 M zinc sulphate until the background turned to a deep white color. Staining was halted by rinsing the gel with deionized water several times. The colorless protein bands of interest were excised using a clean scalpel and incubated 5 min twice into a chelating buffer of 50 mM Tris pH 8, 0.3 M glycine. The bands were rinsed with deionized water twice, crushed to a paste, and incubated at 25 °C with an extraction buffer consisting of 50 mM Tris pH 8 and 0.05% SDS. Extracted protein was recovered from the flow-through using a 100 kDa cut-off filter (Microcon YM-100). The protein content of all samples was determined by SDS-PAGE followed silver nitrate staining and the suitable samples were pooled, concentrated on a 10 kDa cut-off filter (Microcon YM-10), and washed several times with deionized water to remove excess Tris and SDS. The resulting sample (25 μL at 2 μM) was analyzed by ESI-ToF mass spectrometry (LCT, Waters). Twenty microliters of the concentrated sample were manually injected using a gastight syringe into a 20 μl sample loop attached to a Rheodyne injection valve and the loaded onto a C4 trap (Michrom Bioresources). The protein was eluted using a linear gradient from 5 to 95% acetonitrile containing 0.1% formic acid for 15 min at a 36 μl/min flow rate. The theoretical isotopically averaged molecular masses of the proteins and peptides were calculated with the Compute pI/Mw tool (http://web.expasy.org/compute_pi/) (68).

### Lipid bilayer experiments

The pore-forming activity of Rv1698 proteins was analysed in lipid bilayer experiments as described before. Single-channel conductances of the purified MctB proteins were determined using a custom-made lipid bilayer apparatus as described previously (18), (69). Briefly, the Ag/AgCl electrodes of a two-compartment polytetrafluoroethylene cell were bathed in a solution of 1 M KCl, 10 mM Hepes, pH 7.0. Artificial lipid membranes were painted from a solution of 1% diphytanoyl phosphatidylcholine (Avanti Polar Lipids) in *n*-decane. The buffers used for protein storage after purification were examined in lipid bilayer experiments to exclude any channel-forming or membrane-disturbing activity. Current traces for these buffers were recorded for at least three membranes prior protein addition. Then, proteins were added to both sides of the membrane and single-channel conductances for more than 100 pores/sample were digitally recorded. The raw data were analyzed using IGOR Pro program (WaveMetrics) and a macro provided by Dr Harald Engelhardt, and further analyzed in SigmaPlot (Grafiti Software, https://grafiti.com).

### Negative-stain electron microscopy

Approximately 3 μl purified _His6-ΔN26_MctB_tb_ protein (0.1 mg/ml) was deposited onto a glow-discharged carbon-coated 400-mesh Cu EM specimen grid. The sample was stained with 2% (w/w) uranyl formate and air-dried. The data were recorded on a Tecnai G2 F20 TWIN transmission electron microscope (FEI) equipped with a field-emission gun operated at 200 kV. Images were recorded at 71,000× microscope magnification on a 4k × 4k Eagle CCD camera with a pixel size of 1.15 Å per pixel. The defocus ranged from −1.0 to −1.5 μm. A total of 1071 particles were boxed out from 81 micrographs using the e2boxer.py program in EMAN2.1 (70). Reference-free 2D image classification was performed using the EMAN1.9 program refine2d.py.

## Data availability

The dataset(s) supporting the conclusions of this article are included within the article and in the supplemental files. The refined models of the structures of _ΔN26_Mct_Mtb_ crystallized in the presence or the absence of the detergent C_12_E_8_ have been deposited in the Protein Data Bank (http://www.rcsb.org/) with the accession codes 9M9Z and 9MA1, respectively.

## Supporting information

This article contains supporting information (7, 18, 23, 24, 29, 64, 65,74, 75, 76, 77, 78, 79, 80, 81).

## Conflict of interests

The authors declare that they have no conflicts of interest with the contents of this article.
