# Supplementary material for: Oligomerization is required for channel formation of MctB and for copper resistance of Mycobacterium tuberculosis
Source: J Biol Chem. 2025 Dec 9;302(2):111037. doi: 10.1016/j.jbc.2025.111037 (PMC12859517; doi:10.1016/j.jbc.2025.111037)
Supplement: Supplementary Material 1 [file mmc1.pdf]

Supporting Information

Oligomerization is Required for Channel Formation of MctB and for Copper Resistance of *Mycobacterium tuberculosis*

Axel Siroy[1,#], Demeng Sun[2], Avraneel Paul[1], Jennifer L. Rowland[1,$], Lisa M. Jones[1,&], Peter Prevelige[1], Changlin Tian[2] and Michael Niederweis[1*]

Supporting Methods

Construction of plasmids.

E. coli expression vectors for mass/size determination. For the expression in *E. coli* of full-length *rv1698* tagged with a hexa-histidine on its C-terminus, the plasmid pML1020 was constructed by the ligation in the plasmid pET-24(+) digested with restriction nucleases BamHI and XhoI, of the gene *rv1698* amplified by the polymerase-chain reaction (PCR) from genomic *M. tuberculosis* DNA using the oligonucleotides CN1161 and CN1160 and digested with the same enzymes. For the expression in *E. coli* of *rv1698* lacking its N-terminus region and tagged with a hexa-histidine on its C-terminus, the plasmid pML1021 was constructed by the ligation in the plasmid pET-24(+) digested with restriction nucleases BamHI and XhoI, of the truncated gene $_{\Delta 29}$*rv1698* amplified by PCR from genomic *M. tuberculosis* DNA using the oligonucleotides CN1245 and CN1160 and digested with the same enzymes.

Mycobacterial expression vectors. Complementation plasmids pMN035 and pML451, carrying the genes *rv1698* and *ms3747* respectively under the control of P_{SMYC} promoter, were described previously (1,2). The complementation plasmids pML1024 and pML1922, carrying the truncated genes $_{\Delta 7\text{-}29}$*rv1698* and $_{\Delta 7\text{-}29}$*ms3747* respectively under the control of P_{SMYC} promoter, were constructed for this study. The gene encoding $_{\Delta 7\text{-}29}$Rv1698 lacking its N-terminal hydrophobic α-helix was engineered by using a series of PCR amplifications using the plasmid pMN035 as a template: first the upstream region of the gene, including the 6 first codons of *rv1698* was amplified using the oligonucleotides CN145 and CN1370; in parallel, the region coding of the gene starting with the codon for amino-acid $_{30}$Ser was amplified using the oligonucleotides CN1369 and CN1371. Bridging of the fragments was performed by overlap PCR, using the flanking oligonucleotides CN145 and CN1371 and the overlapping one CN1373. The resulting fragment was digested with the restriction endonucleases PacI and SwaI and ligated into the plasmid pMN035 treated with the same enzymes, yielding the plasmid pML1024. The gene encoding $_{\Delta 7\text{-}29}$Ms3747 lacking its N-terminal hydrophobic α-helix was engineered by serial PCR amplification of the gene *ms3747* using the same reverse-backward oligonucleotide CN96 and the forward ones CN1926, CN1927, CN1929 that allowed for the amplification of the gene starting at the amino-acid $_{30}$Ser, the addition of the first 6 codons of *ms3747* and the upstream ribosome binding site as well as a PacI restriction site, respectively. The final PCR product was purified and digested with PacI and KpnI and ligated in the plasmid pML451 digested with the same enzymes, yielding the plasmid pML1922.

Mycobacterial acetamide-inducible expression vectors. We constructed an acetamide-inducible expression vector for the mycobacteria, by cloning the acetamidase operon genes *amiCADS*of *M. smegmatis* in the shuttle vector pMS2: the genes *amiCADS* were amplified by PCR from the plasmid pUGA61B (3) using the oligonucleotides CN1463 and CN1464, introducing the restriction sites for the endonucleases XbaI and NdeI. The resulting fragment was digested using those enzymes and ligated into the plasmid pMS2 digested in the same conditions. The resulting plasmid pML941 therefore lacked any gene in the place of *amiE*, the acetamidase whose expression is regulated by the genes *amiCADS* (4,5), but instead possesses a cloning cassette between the restriction sites NdeI and ClaI. The gene encoding for $MycGFP^{2+}$ was amplified by PCR from the plasmid pMN437 (6) using the oligonucleotides CN97 and CN1460 and subsequently digested with the restrictions enzymes NdeI and ClaI. The resulting fragment was then ligated into the plasmid pML941 digested with the same enzymes, yielding the plasmid

pML943. The gene encoding $MctB_{sm}$ (*msmeg_3747*) was amplified by PCR using the oligonucleotides CN1458 and CN1469 (phosphorylated), the resulting fragment was treated with NdeI to yield a compatible 5' end with the plasmid pML943 digested with NdeI and SwaI or NdeI and EcoRV where it was ligated with, yielding the plasmid pML948 and pML946, respectively.
The gene coding for $_{\Delta 7\text{-}29}$Ms3747 lacking its N-terminal hydrophobic α-helix was amplified by PCR from the plasmid pML1922 using the oligonucleotides CN1928 and CN1469 and the resulting fragment was digested with the restriction endonucleases NdeI and KpnI and ligated in the plasmids pML948 or pML946 treated by the same enzymes, yielding the plasmids pML1921 and pML1920, respectively.

Mycobacterial SS_{mspA}-mctB constructs. The Sec signal sequence of MspA (residues 1-34) was fused to MctB lacking amino acids residues 1-29 (MctB 30-284) with ‘BlaTEM1 at the C-terminus of MctB. The DNA construct was generated by overlapping PCR. The smyc promoter and DNA encoding the MspA signal sequence (*SS_{mspA}*) were amplified from pML2166 using primers CN2016 and CN3274 (creates the overlap with *SS_{mspA}* and $_{\Delta 29}$*ms3747*). MctB lacking its N-terminal hydrophobic helix ($_{\Delta 29}$*ms3747*) fused to ‘BlaTEM1 (*‘blaTEM1*) was amplified from pML1948 using primers CN3273 (creates the overlap with *SS_{mspA}* and $_{\Delta 29}$*ms3747*) and CN0131. The two PCR products were purified, mixed and the final single construct was amplified using CN2016 and CN0131. The product was digested with XbaI and HindIII and ligated into pMN016 digested with the same enzymes, resulting in plasmid pML3800. Additionally, the MspA signal sequence was added to $_{\Delta 29}$MctB without ‘BlaTEM1. The smyc promoter and MspA signal sequence were amplified as for pML3800, the $_{\Delta 29}$*mctB* fragment was amplified with CN3273 and CN0131 from pML451; the two PCR products were purified, mixed, and the full-length product was amplified using primers CN2016 and CN0131. The final PCR product was digested with XbaI and HindIII and ligated into pMN016 digested with the same enzymes resulting in pML3801.

E. coli expression vectors for crystallisation of 6xHis-$MctB_{tb}$ and 6xHis-$_{\Delta N26}MctB_{tb}$. A truncated $MctB_{tb}$ lacking its first 26 amino acids (residues 27-314, denoted 6xHis- $_{\Delta N26}MctB_{tb}$,) was expressed and purified as previously described (7). Briefly, the gene encoding $_{\Delta N26}MctB_{tb}$ was amplified by PCR from a DNA library of *M. tuberculosis*, using the primer ΔN26-fw and ΔN26-bw pair. The amplicon and the plasmid pET-28a (Novagen) were digested with the enzymes NdeI and XhoI then ligated together to yield the expression vector p28-$Rv1698_{\Delta N26}$ in which the sequence coding for a hexa-histidine tag is translationally fused to the new N-terminus of $_{6His}rRv1698_{\Delta 26}$.
The vector used for the production of full-length recombinant $MctB_{tb}$ was constructed in the same manner, using the oligonucleotide pair FL-fw and ΔN26-bw pair for the PCR step, resulting in the plasmid p28-$Rv1698_{FL}$ in which the sequence coding for a hexa-histidine tag is translationally fused to the new N-terminus of 6xHis-$MctB_{tb}$.

Supporting Figures

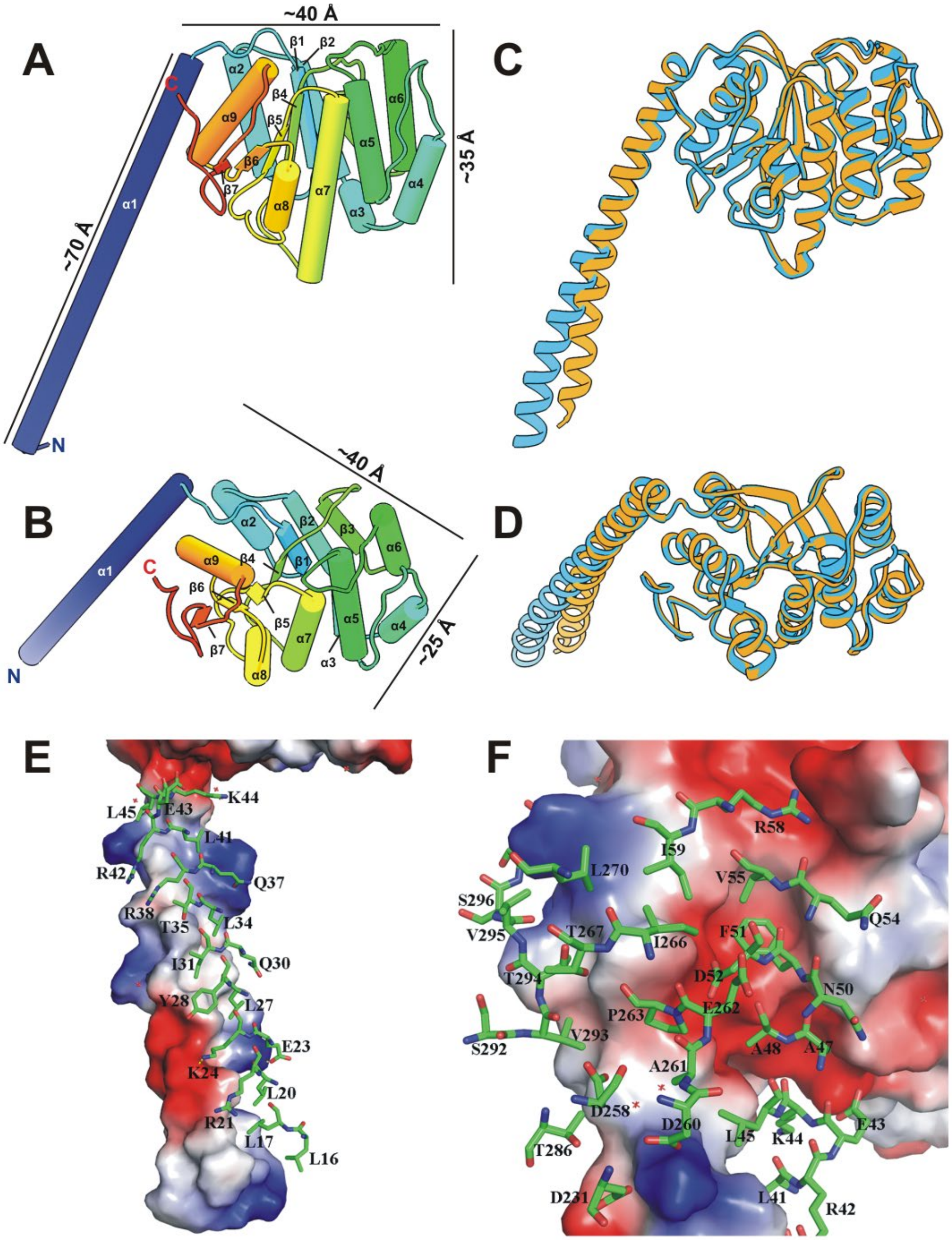

Figure S1. Topological organization of the monomer of $_{\Delta N26}$MctB$_{tb}$ seen from the side **(A)** or the top of the molecule **(B)**. The α-helices and β-strands assigned in the topological model (Fig. 4B) are indicated as well as the dimensions. Structural overlap of the two monomers of $_{\Delta N26}$MctB$_{tb}$ purified and crystallized in the presence of $C_{12}E_8$ as seen from the side **(C)** or the top **(D)** of the molecules. Orange: chain A; Cyan: chain **C.** Detail of the dimeric interface of the two $_{\Delta N26}$MctB$_{tb}$ molecules in one crystal asymmetric unit in the N-terminal long coiled-coil region **(E)** and the central globular domain region **(F)**.

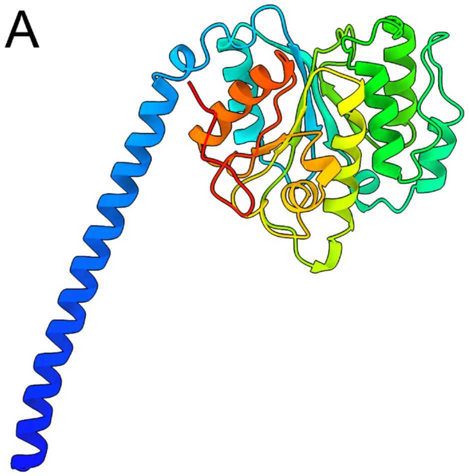

	AF3 monomer		AF3 dimer	
	**ΔN26 (C)**	**+N26 (D)**	**ΔN26 (E)**	**+N26 (F)**
Best pairs (RMSD ≤2Å)	0.73Å (222 pairs)	0.84Å (260 pairs)	0.81Å (233 pairs)	0.92Å (270 pairs)
Global (all pairs)	2.60Å (288 pairs)	2.45Å (289 pairs)	1.26Å (288 pairs)	1.20Å (289 pairs)

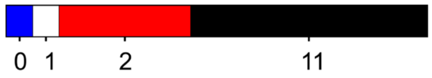

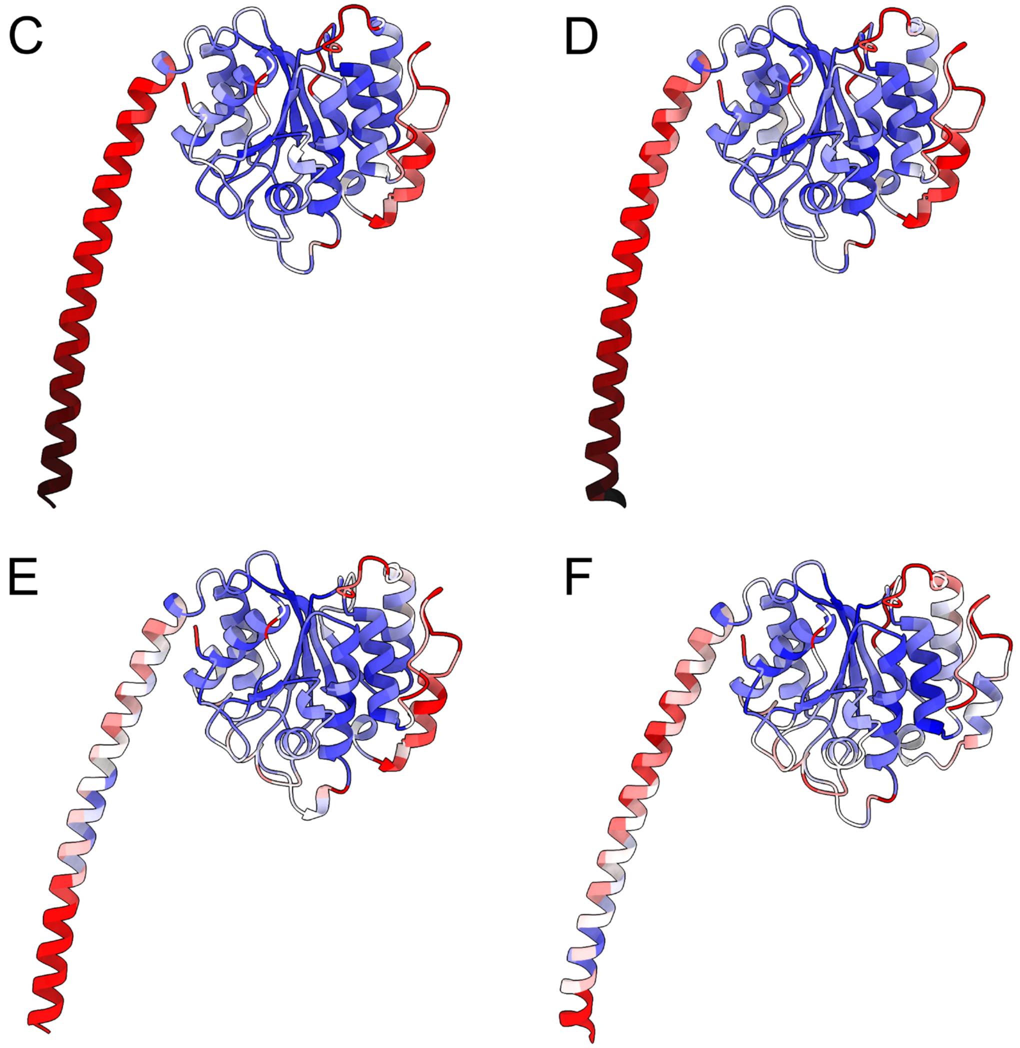

Figure S2. Comparison of the crystal structure of the MctB monomer with a predictive model generated by AlphaFold.

Predictive models of the protein Rv1698 (Uniprot P9WJ83) were produced with AlphaFold 3 (AF3) and compared to the structural model derived by X-ray analysis of Rv1698 crystals in the presence of the detergent $C_{12}E_8$ (**A**, rainbow-colored from glycine 27 in blue to asparagine 314 in red). The monomeric chains derived from the predicted models were superimposed with the MatchMaker tool in ChimeraX 1.7, to the chain C of the experimentally validated model, and the Root Mean Square Deviations (RMSD) from the Cα chain were measured and depicted on the different models, ranging from 0.04 Å (blue) to over 11 Å (black); the pruning cutoff value of 2 Å is shown in red. **C**. AF3 model of monomeric Rv1698, lacking its 26 N-terminal residues ($_{\Delta N26}$MctB$_{tb}$). **D**. AF3 model of full-length monomeric Rv1698, depicted without its 26 N-terminal residues (MctB$_{tb}$). **E**. Single chain from the AF3 model of dimeric Rv1698, lacking its 26 N-terminal residues ($_{\Delta N26}$MctB$_{tb}$). **F**. Single chain from the AF3 model of full-length dimeric Rv1698, depicted without its 26 N-terminal residues (MctB$_{tb}$). RMSD values for the full alignment, as well as the best conserved regions (below the RMSD cut-off value of 2 Å) are reported in the panel **B**.

1 MISLRQHAVS LAAVFLALAM GVVLGSGF**FS DTLLSSLRSE KRDLYTQIDR LTDQRDALRE KLSAADNFDI**

71 **QVGSRIVHDA LVGKSVVIFR TPDAHDDDIA AVSKIVGQAG GAVTATVSLT QEFVEANSAE KLRSVVNSSI**

141 **LPAGSQLSTK LVDQGSQAGD LLGIALLSNA DPAAPTVEQA QRDTVLAALR ETGFITYQPR DRIGTANATV**

211 **VVTGGALSTD AGNQGVSVAR FAAALAPRGS GTLLAGRDGS ANRPAAVAVT RADADMAAEI STVDDIDAEP**

281 **GRITVILALH DLINGGHVGH YGTGHGAMSV TVSQHHHHHH**

Figure S3. Analysis of full-length MctB by mass spectroscopy.
The amino acids shown in bold in the sequence of MctB of *M. tuberculosis* (Rv1698-6xHis) were identified by peptide mass fingerprinting using MALDI-ToF and ESI-FT-ICR. Potential trypsin cleavage sites are indicated by black squares (R, K). The underlined sequence indicates the peptide closest to the N-terminus identified by those techniques.

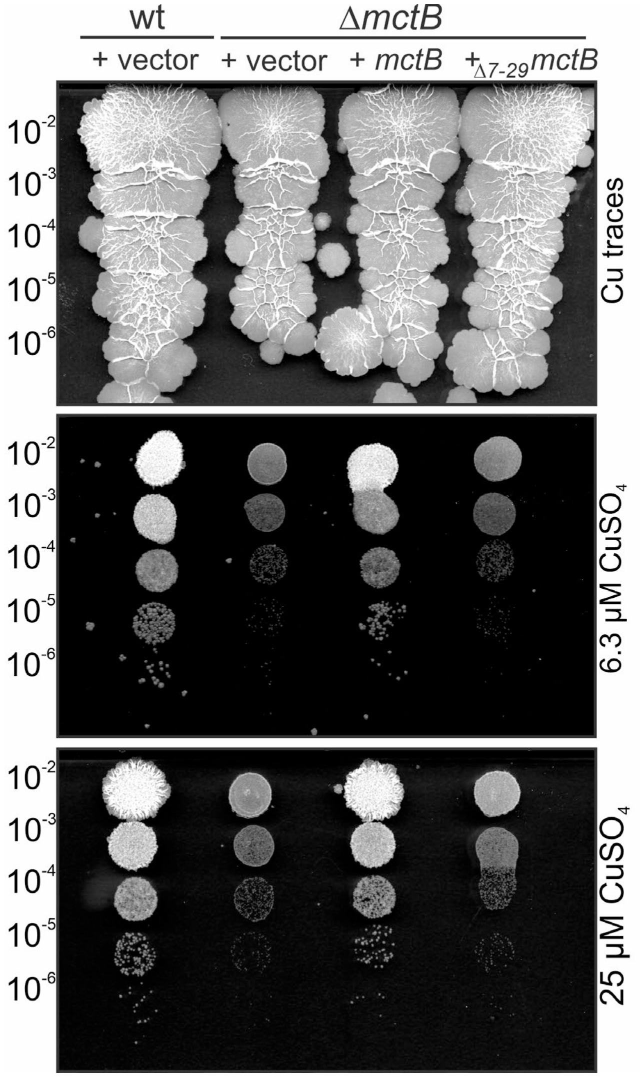

Figure S4. Role of the N-terminus of the *M. tuberculosis* MctB protein in copper sensitivity of *M. smegmatis.*

Growth of the indicated *M. smegmatis* strains on agar plates containing different copper concentrations. The strains are wt *M. smegmatis* containing the vector control pMS2 and the *ΔmctB* mutant ML77 carrying the plasmids pMS2, pMN035 (p_{smyc}::*rv1698*) and pML1024 (p_{smyc}::$_{\Delta 7-29}$*rv1698*) producing wt MctB and the truncated $_{\Delta 7-29}$MctB proteins, respectively.

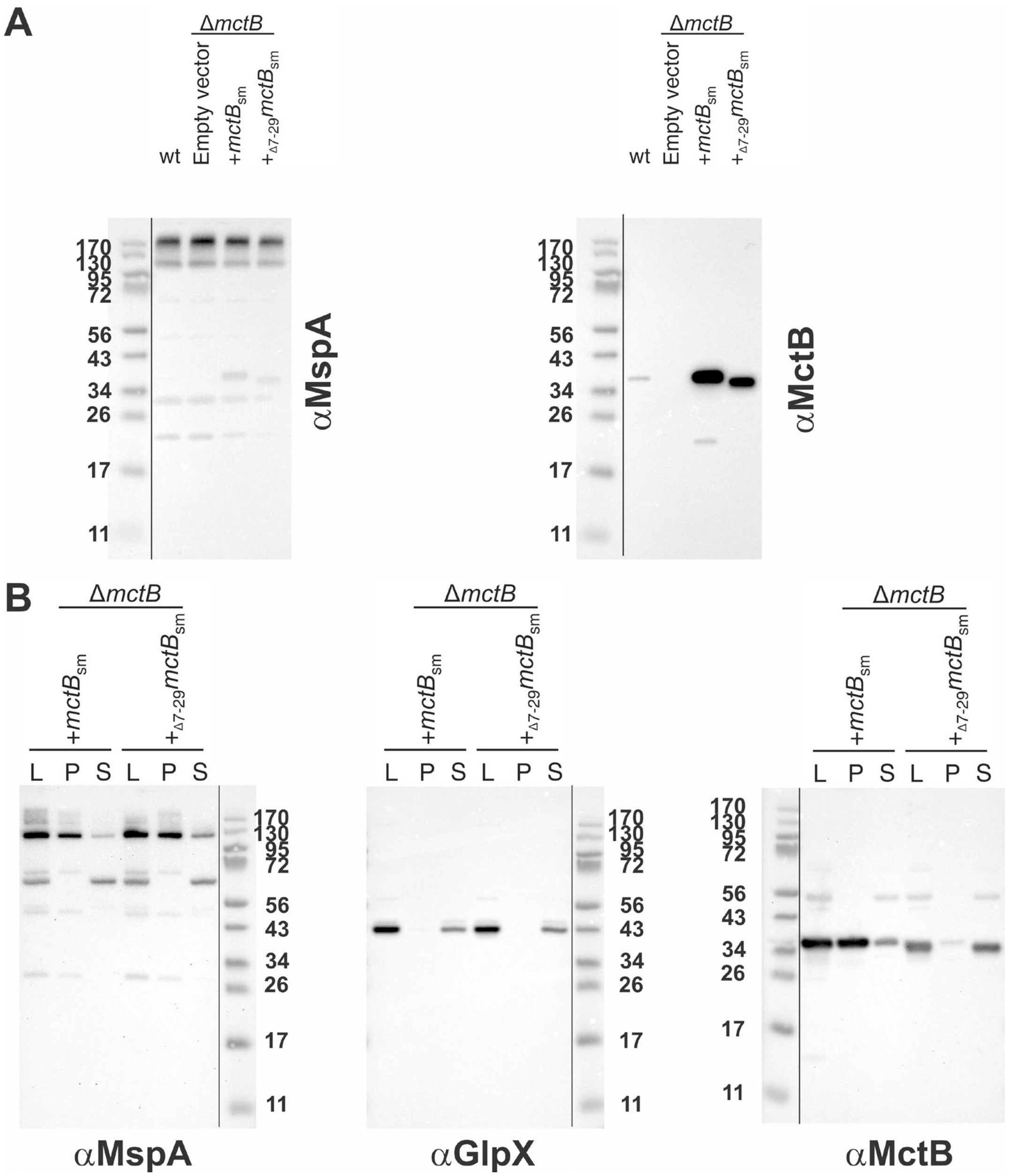

Figure S5. Role of the N-terminus in the subcellular localization of MctB.
A. Comparison of protein levels of full-length MctB and $_{\Delta7\text{-}29}$MctB$_{sm}$ in *M. smegmatis* ML77 compared to wild-type protein levels (SMR5) by Western blot analysis using the monoclonal antibody 5D1.23. MspA, was used as a protein loading control. **B.** Western blot of sub-cellular fractions of cell lysates (L) of *M. smegmatis* ML77 (Δ*mctB*) expressing full-length MctB$_{sm}$ or $_{\Delta7\text{-}29}$MctB$_{sm}$. Soluble proteins (S) were recovered in the supernatant while the cell envelope proteins were recovered in the membrane pellet (P). GlpX (Rv1099, Fructose 1,6-bisphosphatase) and MspA were used as cytosolic and membrane loading controls, respectively.
It should be noted that the electrophoretic mobility of the main MspA band in SDS extracts of whole *M. smegmatis* cell lysates shifts from >170 kDa (Fig. S6A) to ~120 kDa (Fig. S6A) in subcellular fractions due to reduced incubation times at 95 °C.
The images are full-length Western blots shown partially in figures 3B and 3C.

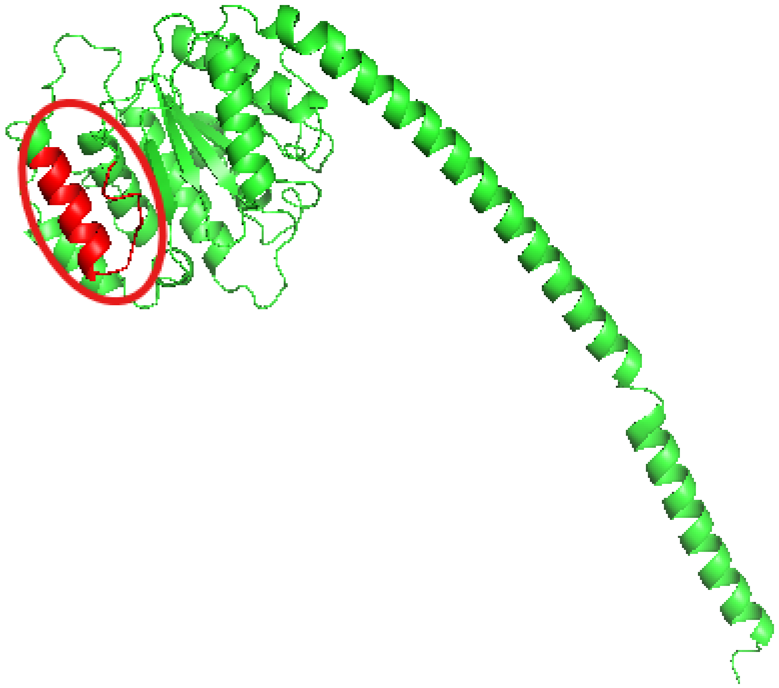

MISLRQHAVSLAAVFLALAMGVVLGSGFFSDTLLSSLRSEKRDLYTQIDRLTDQRDALR
EKLSAADNFDIQVGSRIVHDALVGKSVVIFRTPDAHDDDIAAVSKIVGQAGGAVTATVSL
TQEFVEANSAEKLRSVVNSSILPAGSQLSTKLVDQGSQAGDLLGIALLSNADPAAPTVE
QAQRDTVLAALRETGFITYQPRDRIGTANATVVVTGGALSTDAGNQGVSVARFAAALA
PRGSGTLLAGRDGSANRPAAVAVTRADADMAAEISTVDDIDAEPGRITVILALHDLING
GHVGHYGTGHGAMSVTVSQ

Figure S6. Epitope of MctB recognized by the monoclonal antibody 5D1.23.
The epitope of the monoclonal antibody 5D1.23 was determined by peptide mapping (Wolschendorf et al., unpublished) and matched the MctB amino acid sequence shown in red. The epitope is highlighted in red in the structure of monomeric MctB (Rv1698).

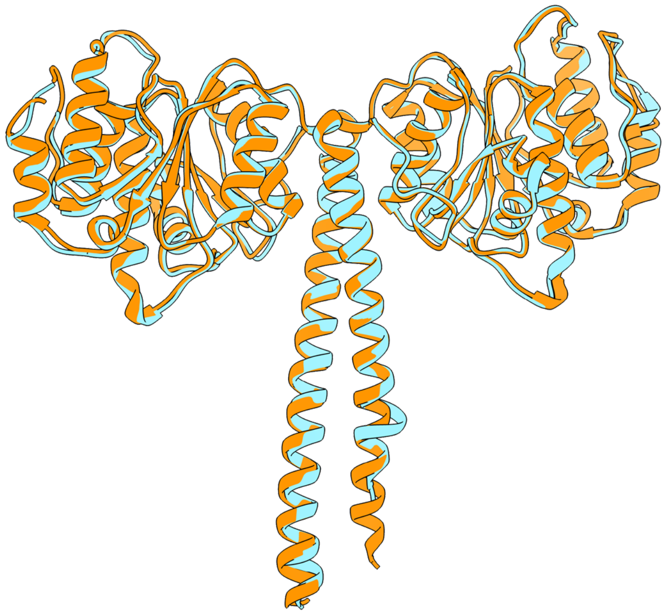

Figure S7. The structure of MctB is not altered by detergents.
Comparison of the structures of $_{\Delta N26}$MctB$_{tb}$ purified and crystallized in the absence (cyan) or the presence of the detergent $C_{12}E_8$ (orange). The pair-wise RMSD value of the two structures is 0.455 Å for 516 Cα atomic pairs.

A

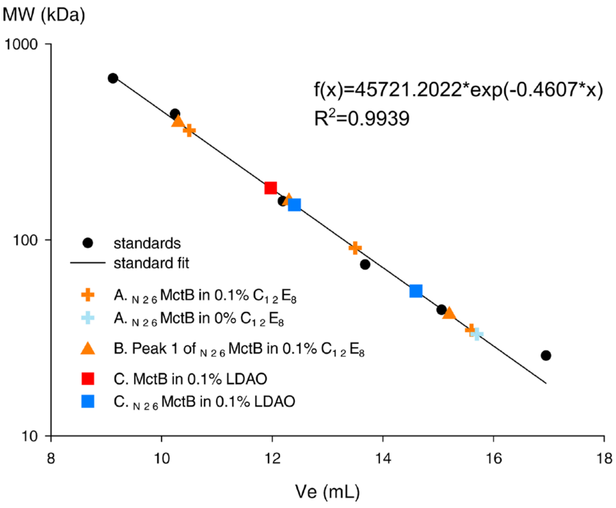

B

		Ve (mL)	MW (kDa)
**MW standards**	Thyroglobulin	9.1	669.0
	Ferritin	10.2	440.0
	Aldolase	12.2	158.0
	Conalbumin	13.7	75.0
	Ovalbumin	15.1	44.0
	Chymotrypsinogen	17.0	25.7
**Fig. 5A**			
$_{N26}$MctB in 0.1% $C_{12}E_8$	Peak 1	10.5	362.5
	Peak 2	13.5	91.0
	Peak 3	15.6	34.6
$_{N26}$MctB in 0% $C_{12}E_8$	Peak	15.7	33.0
**Fig. 5B**			
Peak 1 $_{N26}$MctB in 0.1% $C_{12}E_8$	Peak 1	10.3	397.5
	Peak 2	12.3	158.2
	Peak 3	15.2	41.6
**Fig. 5C**			
MctB in 0.1% LDAO	Peak	12.0	184.1
$_{N26}$MctB in 0.1% LDAO	Peak 1	12.4	151.1
	Peak 2	14.6	54.8

Figure S8. Calibration of the gel filtration column used for separation of MctB oligomers. The Superdex200 10/300 column was calibrated with Bio-Rad molecular mass standards, plotted in SigmaPlot 12.5 (Systat Software) and a nonlinear regression (exponential decay) was calculated. The elution volumes (Ve) of the different peaks of MctB were plotted on the calibration curve (A) and the calculated molecular masses are reported in the adjacent table (B).

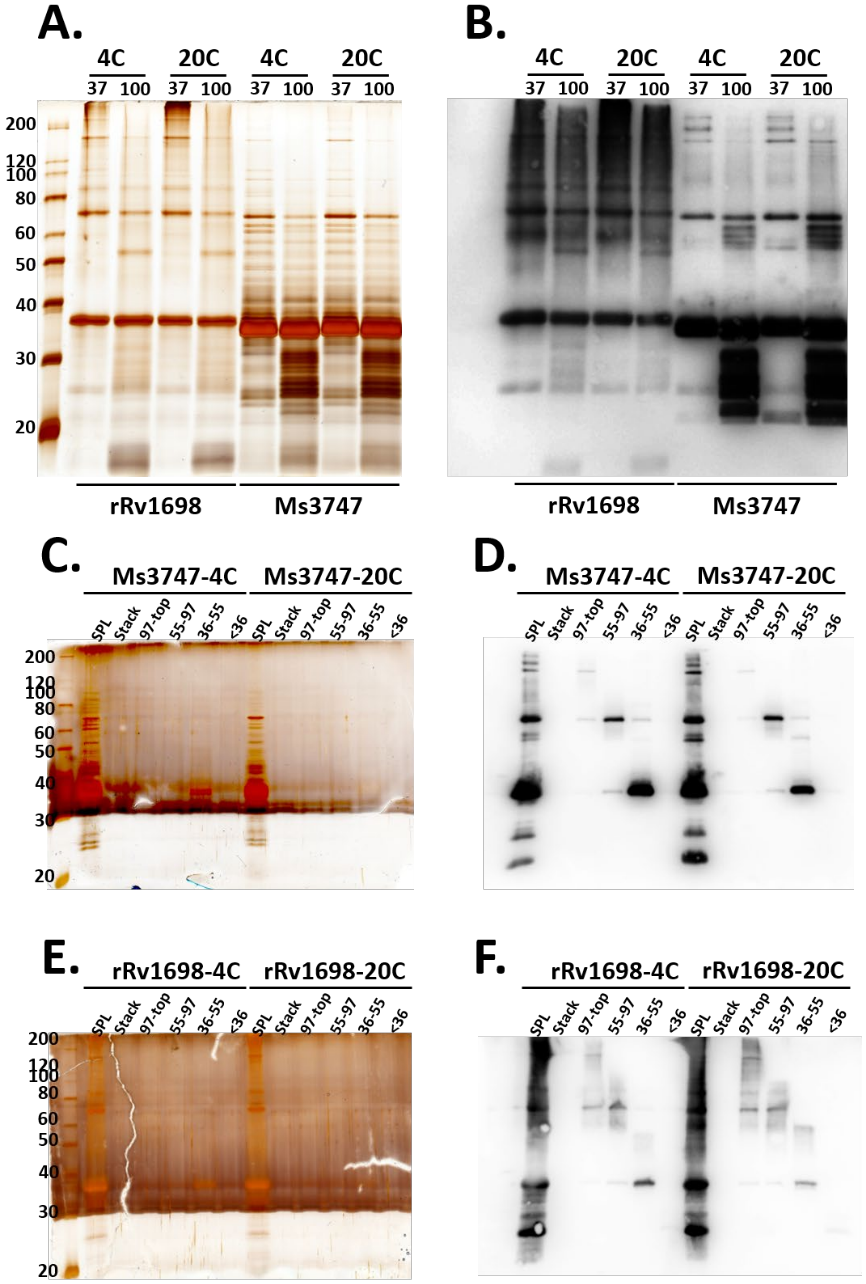

Figure S9. Gel electrophoretic separation and isolation of monomeric and oligomeric MctB. Freshly purified batches of MctB produced recombinantly in *E. coli* (rRv1698) or in *M. smegmatis* (Ms3747) were stored at 4°C or room temperature (20°C) for a week before analysis by SDS-PAGE (**A**) in mild conditions (0.1% SDS in the loading dye, no reducing agent, pre-warming of the sample at 37°C or heating to 100°C, cold-bath electrophoresis), stained with silver nitrate, and analyzed by Western blots (**B**) using the monoclonal anti-MctB antibody 5D1-23. Following this analysis, the different oligomer forms were extracted from sections of preparative electrophoresis gels (same conditions, no heat denaturation) using an OPOE-containing buffer. SDS-PAGE and Western blot analyses were performed on the purified Ms3747 and rRv1698 oligomers (panels **C** and **D**, panels **E** and **F**, respectively). Panel D (4 °C) shows the same Western blot as in Fig. 5D.

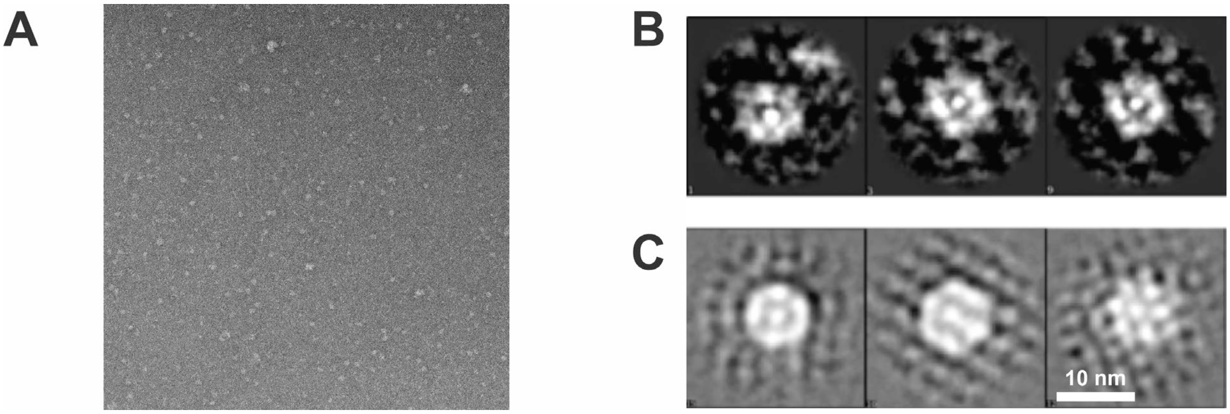

Figure S10. Electron microscopy analysis of MctB.
A. Electron micrograph of purified *M. tuberculosis* MctB oligomers isolated in LDAO after negative staining with uranyl acetate. **B.** Selected raw electron micrographs of single MctB particles. **C.** Class-averaged particles as determined from the electron micrographs.

Supporting Tables

Strain	Parent strain and relevant genotype	Source or reference
E. coli DH5α	F^- *rec*A1, *end*A1, *gyr*A96, *thi*; *rel*A1, *hsd* R17(r_K^-,m_K^+), *sup*E44, Φ80d *lacZ* ΔM15, Δ(*lacZYA-argF*) UE169	(9)
E. coli BL21(DE3)	F^- *ompT hsd S*$_B$ ($r_B^-m_B^-$) *gal dcm lacYI* , (DE3)	
E. coli Rosetta	F^- *ompT hsd S*$_B$($r_B^-m_B^-$) *gal dcm lacYI* pRARE (Cam^R), (DE3)	(10)
E. coli B834(DE3)	F^- *ompT hsd S*$_B$ ($r_B^-m_B^-$) *gal dcm met*, (DE3)	(11)
M. smegmatis SMR5	*M. smegmatis* mc^2155; Sm^R	(12)
M. smegmatis ML77	SMR5 derivative, Δ*mctB::loxP*	(2)
M. smegmatis PM759	*ept-1* Δ*lysA4 rpsL6* ; Δ*blaS1*	(13)
M. bovis BCG	Pasteur 35739	ATCC

Table S1: Strains used in this work.
The annotations Sm^R, Amp^R, Cam^R, Hyg^R and Kan^R indicate resistance to the antibiotics streptomycin, ampicillin, chloramphenicol, hygromycin and kanamycin, respectively.

Plasmid	Parent vector, relevant genotype and properties	Origin
pET-24(+)	pBR322 origin, f1 origin,lacI, p_{T7} , *his_6* **,** Kan^R	Novagen
pUGA61B	pMV306, p_{ACET}::*torA-gfp*; Kan^R	(3)
pMS2	ColE1 origin, PAL5000 origin; Hyg^R	(14)
pMN016	p_{SMYC}-*mspA;* ColE1 origin; PAL5000 origin; Hyg^R	(15)
pMN035	p_{SMYC}-*rv1698;* ColE1 origin; PAL5000 origin; Hyg^R	(1)
pMN437	p_{SMYC}-*$gfp_m{}^{2+}$;* ColE1 origin; PAL5000 origin; Hyg^R	(6)
pML451	p_{SMYC}-*ms3747;* ColE1 origin; PAL5000 origin; Hyg^R	(1)
pML911	p_{SMYC}-*rv1698*-His_6*;* ColE1 origin; PAL5000 origin; Hyg^R	(1)
pML941	p_{ACET}-ColE1 origin; PAL5000 origin; Hyg^R	This study
pML943	p_{ACET}-*$gfp_m{}^{2+}$;* ColE1 origin; PAL5000 origin; Hyg^R	This study
pML946	p_{ACET}-*ms3747-$gfp_m{}^{2+}$;* ColE1 origin; PAL5000 origin; Hyg^R	This study
pML948	p_{ACET}-*ms3747;* ColE1 origin; PAL5000 origin; Hyg^R	This study
pML1020	p_{T7}-*rv1698*-His_6, Kan^R	This study
pML1021	p_{T7}-*$_{\Delta N28}$rv1698*-His_6, Kan^R	This study
pML1024	p_{SMYC}-*$_{\Delta 7\text{-}29}$rv1698;* ColE1 origin; PAL5000 origin; Hyg^R	This study
pML1920	p_{ACET}-*$_{\Delta 7\text{-}29}$ ms3747-$gfp_m{}^{2+}$;* ColE1 origin; PAL5000 origin; Hyg^R	This study
pML1921	p_{ACET}-*$_{\Delta 7\text{-}29}$ ms3747;* ColE1 origin; PAL5000 origin; Hyg^R	This study
pML1922	p_{SMYC}-*$_{\Delta 7\text{-}29}$ms3747;* ColE1 origin; PAL5000 origin; Hyg^R	This study
pML1930	p_{SMYC}-*$_{\Delta 7\text{-}29}$ ms3747-$gfp_m{}^{2+}$;* ColE1 origin; PAL5000 origin; Hyg^R	This study
pML1931	p_{SMYC}-*$_{\Delta 7\text{-}29}$ ms3747;* ColE1 origin; PAL5000 origin; Hyg^R	This study
pML2165	p_{SMYC}-*blaTEM-1;* ColE1 origin; PAL5000 origin; Hyg^R	This study
pML2166	p_{SMYC}-*ss_{mspA}'blaTEM-1;* ColE1 origin; PAL5000 origin; Hyg^R	This study
pML2167	p_{SMYC}-*'blaTEM-1;* ColE1 origin; PAL5000 origin; Hyg^R	This study
pML1948	p_{SMYC}-*ms3747'blaTEM-1;* ColE1 origin; PAL5000 origin; Hyg^R	This study
pML1949	p_{SMYC}-*$_{\Delta 7\text{-}29}$ ms3747'blaTEM-1;* ColE1 origin; PAL5000 origin; Hyg^R	This study
pML3800	p_{SMYC}- *ss_{mspA} -$_{\Delta 7\text{-}29}$ ms3747'blaTEM-1;* ColE1 origin; PAL5000 origin; Hyg^R	This study
pML3801	p_{SMYC}- *ss_{mspA} -$_{\Delta 7\text{-}29}$ ms3747;* ColE1 origin; PAL5000 origin; Hyg^R	This study

Table S2: Plasmids used in this work.

The annotations Sm^R, Amp^R, Cam^R, Hyg^R and Kan^R indicate resistance to the antibiotics streptomycin, ampicillin, chloramphenicol, hygromycin and kanamycin, respectively. *mspA* is the main porin gene of *M. smegmatis*. MctB contains a 29 amino acid leader sequence encoded by the first 87 nucleotide bases in *rv1698* or *ms3747* in *M. tuberculosis* or *M. smegmatis* respectively. Tags of 6 consecutive histidine residues are referred to as 6xHis, and the position of the tag (N- or C-terminal) is indicated by its position in the name. PAL5000 is the mycobacterial origin of replication.

Oligonucleotide	Sequence (5' → 3')
CN96	CGTTCTCGGCTCGATGATCC
CN97	TTACACATGACCAACTTCGATAACG
CN145	CGACCAGCACGGCATACATC
CN924	AAACTGCTCTCGGTGGTCAAC
[1]CN1160	ATCTCGAGCTA**GTGGTGGTGGTGGTGGTG**CTGGGAAACCGTGACTGACATCGC
[2]CN1161	CAGGATCCAAGAAGGAGATATACCATGATCTCGTTGCGTCAAC
CN1245	CAGGATCCAAGAAGGAGATATACCATGTTCTCCGATACTTTGCTGTCC
CN1369	TCCGATACTTTGCTGTCCAGCTTGCGT
CN1370	TTGACGCAACGAGATCATATTAACCTCCT
[3]CN1371	CTTATTTAAATGGAACACGCCCTAACGCGGGCCTA
CN1373	GCAAGCTGGACAGCAAAGTATCGGATTGACGCAACGAGATCATATTAACCTCCT
[4]CN1458	AATTTACATATGATAACGCTACGGGCGCAC
[4]CN1460	TTTAATCATATGTCGAAGGGCGAGGAGCTGTTCAC
[4]CN1463	ATCATTTAAATAGTACTCATATGGACTCCCTTTCTCTTATCG
[5]CN1464	AAACTCTAGAGAAGTGACGCGGTCTCAAGCGTC
CN1469	ATCCTGCGGGACCGTCACCGAAGAC
[6]CN1734	CGTTAATTAAAGAAAGGAGGTTAATATGAGTATTCAACATTTCCG
CN1926	TCCAACACCGTGCTGTCGGGTCTG
CN1927	TAATCTATGATAACGCTACGGGCGTCCAACACCGTGCTGTCGGG
[4]CN1928	AAAGGGAGTCCATATGATAACGCTACGGGCGTCCAACACCGTGCTGTCGGG
CN1929	GCATGCTTAATTAAGCAGAAAGGAGGTTAATCTATGATAACGCTACG
CN2193	CAGCGTTTCTGGGTGAGCGCTCAGCTCGTTGTCCAG
CN2195	CTGGACAACGAGCTGAGCGCTCACCCAGAAACGCTG
[6]CN2196	CGTTAATTAAAGAAAGGAGGTTAATATGGCTCACCCAGAAACGCTGG
[7]CN3053	TAAAGCTTTAGTGGTGGTGGTGGTGGTGAGTACTGGCGTAGTCCGGCACGTCGTACGGGT AGATATCCCAATGCTTAATCAGTGAGG
CN3207	TACCCGTACGACGTGCCGGACTACGCCAGTAACGCTCACCCAGAAACGCTGGTG
CN3208	CTTCGGTGACGGTCCCGCAGGATATCTACCCGTACGACGTGCCGGACTACGCC
CN0131	CTCTAGGGTCCCCAATTAATTAGC
CN2016	CCGATTCATTAATGCAGCTAGAACTAG
CN3273	GCCTGGACAACGAGCTGAGCTCCAACACCGTGCTGTCGGGTCTG
CN3274	CAGACCCGACAGCACGGTGTTGGAGCTCAGCTCGTTGTCCAGGC
[4]ΔN26-fw	CGAATCATATGGGCTTTTTCTCCGATACTTTGCTGT
[1]ΔN26-bw	CAGCTCGAGTTACTGGGAAACCGTGACTGACATCG
[4]FL-fw	AGGAATTCCATATGATCTCGTTGCGTCAACATG

Table S3: Oligonucleotides used in this work.
Restriction sites used for cloning are underlined: [1]XhoI, [2]BamHI, [3]SwaI, [4]NdeI, [5]XbaI, [6]PacI, [7]HindIII. The hexa-histidine tag coding sequence is in bold.

Peptide	MH+	Sequence	Position
1	1138.61	FSDTLLSSLR(S)	29 - 38
2	1523.78	(R)SEKRDLYTQIDR(L)	39 - 50
3*	1179.61	(K)RDLYTQIDR(L)	42 - 50
4	1792.93	(K)RDLYTQIDRLTDQR(D)	42 - 55
5*	1023.51	(R)DLYTQIDR(L)	43 - 50
6	1636.83	(R)DLYTQIDRLTDQR(D)	43 - 55
7	2092.08	(R)DLYTQIDRLTDQRDALR(E)	43 - 59
8	2205.13	(R)DALREKLSAADNFDIQVGSR(I)	56 - 75
9*	1749.88	(R)EKLSAADNFDIQVGSR(I)	60 - 75
10	2682.42	(R)EKLSAADNFDIQVGSRIVHDALVGK(S)	60 - 84
11*	1492.74	(K)LSAADNFDIQVGSR(I)	62 - 75
12	2425.28	(K)LSAADNFDIQVGSRIVHDALVGK(S)	62 - 84
13*	951.56	(R)IVHDALVGK(S)	76 - 84
14	3088.64	(R)IVHDALVGKSVVIFRTPDAHDDDIAAVSK(I)	76 - 104
15*	720.44	(K)SVVIFR(T)	85 - 90
16*	2156.10	(K)SVVIFRTPDAHDDDIAAVSK(I)	85 - 104
17*	2677.37	(K)IVGQAGGAVTATVSLTQEFVEANSAEK(L)	105 - 131
18	2946.55	(K)IVGQAGGAVTATVSLTQEFVEANSAEKLR(S)	105 - 133
19	4615.46	(K)IVGQAGGAVTATVSLTQEFVEANSAEKLRSVVNSSILPAGSQLSTK(L)	105 - 150
20*	1957.11	(K)LRSVVNSSILPAGSQLSTK(L)	132 - 150
21*	1687.92	(R)SVVNSSILPAGSQLSTK(L)	134 - 150
22	4887.57	(R)SVVNSSILPAGSQLSTKLVDQGSQAGDLLGIALLSNADPAAPTVEQAQR(D)	134 - 182
23*	3218.67	(K)LVDQGSQAGDLLGIALLSNADPAAPTVEQAQR(D)	151 - 182
24	4058.15	(K)LVDQGSQAGDLLGIALLSNADPAAPTVEQAQRDTVLAALR(E)	151 - 190
25*	858.50	(R)DTVLAALR(E)	183 - 190
26*	2051.09	(R)DTVLAALRETGFITYQPR(D)	183 - 200
27	2322.22	(R)DTVLAALRETGFITYQPRDR(I)	183 - 202
28*	1211.61	(R)ETGFITYQPR(D)	191 - 200
29	1482.73	(R)ETGFITYQPRDR(I)	191 - 202
30	4050.06	(R)ETGFITYQPRDRIGTANATVVVTGGALSTDAGNQGVSVAR(F)	191 - 230
31*	2857.48	(R)DRIGTANATVVVTGGALSTDAGNQGVSVAR(F)	201 – 230
32	3654.93	(R)DRIGTANATVVVTGGALSTDAGNQGVSVARFAAALAPR(G)	201 – 238
33*	2586.35	(R)IGTANATVVVTGGALSTDAGNQGVSVAR(F)	203 – 230
34*	816.47	(R)FAAALAPR(G)	231 – 238
35	1628.92	(R)FAAALAPRGSGTLLAGR(D)	231 – 247
36*	831.47	(R)GSGTLLAGR(D)	239 – 247
37*	2197.18	(R)GSGTLLAGRDGSANRPAAVAVTR(A)	239 – 261
38*	1384.73	(R)DGSANRPAAVAVTR(A)	248 – 261
39	3527.67	(R)DGSANRPAAVAVTRADADMAAEISTVDDIDAEPGR(I)	248 – 282
40	6219.96	(R)ADADMAAEISTVDDIDAEPGRITVILALHDLINGGHVGHYGTGHGAMSVTVSQHHHHHH	262 – 320

Table S4. Peptide mass fingerprinting analysis of $MctB_{HIS6}$.

MctBHis6 was purified from M. bovis BCG and analyzed by Electrospray Ionization Fourier Transform Ion Cyclotron Resonance Mass Spectrometry (ESI FT-ICR MS). The identified peptides are ordered according to their position in the sequence of Rv1698. The masses displayed correspond to the monocharged species (MH^+) of each ion. Asterisks indicate peptides further identified by MALDI-ToF analysis. Amino acids shown in parentheses flank the identified peptides.

REFERENCES

1. Siroy, A., Mailaender, C., Harder, D., Koerber, S., Wolschendorf, F., Danilchanka, O., Wang, Y., Heinz, C., and Niederweis, M. (2008) Rv1698 of *Mycobacterium tuberculosis* represents a new class of channel-forming outer membrane proteins. *J. Biol. Chem.* **283**, 17827-17837
2. Wolschendorf, F., Ackart, D., Shrestha, T. B., Hascall-Dove, L., Nolan, S., Lamichhane, G., Wang, Y., Bossmann, S. H., Basaraba, R. J., and Niederweis, M. (2011) Copper resistance is essential for virulence of *Mycobacterium tuberculosis*. *Proc Natl Acad Sci U S A* **108**, 1621-1626
3. Posey, J. E., Shinnick, T. M., and Quinn, F. D. (2006) Characterization of the twin-arginine translocase secretion system of *Mycobacterium smegmatis*. *J Bacteriol* **188**, 1332-1340
4. Parish, T., Mahenthiralingam, E., Draper, P., Davis, E. O., and Colston, M. J. (1997) Regulation of the inducible acetamidase gene of Mycobacterium smegmatis. *Microbiology* **143 (Pt 7)**, 2267-2276
5. Triccas, J. A., Parish, T., Britton, W. J., and Gicquel, B. (1998) An inducible expression system permitting the efficient purification of a recombinant antigen from *Mycobacterium smegmatis*. *FEMS Microbiol Lett* **167**, 151-156
6. Steinhauer, K., Eschenbacher, I., Radischat, N., Detsch, C., Niederweis, M., and Goroncy-Bermes, P. (2010) Rapid evaluation of the mycobactericidal efficacy of disinfectants in the quantitative carrier test EN 14563 by using fluorescent *Mycobacterium terrae*. *Appl Environ Microbiol* **76**, 546-554
7. Chen, L., Sun, D., Wu, M., Zang, J., and Tian, C. (2010) Cloning, expression, purification, crystallization and preliminary crystallographic analysis of Rv1698, an outer membrane channel protein from Mycobacterium tuberculosis. *Acta Crystallogr Sect F Struct Biol Cryst Commun* **66**, 1525-1527
8. Abramson, J., Adler, J., Dunger, J., Evans, R., Green, T., Pritzel, A., Ronneberger, O., Willmore, L., Ballard, A. J., Bambrick, J., Bodenstein, S. W., Evans, D. A., Hung, C. C., O'Neill, M., Reiman, D., Tunyasuvunakool, K., Wu, Z., Zemgulyte, A., Arvaniti, E., Beattie, C., Bertolli, O., Bridgland, A., Cherepanov, A., Congreve, M., Cowen-Rivers, A. I., Cowie, A., Figurnov, M., Fuchs, F. B., Gladman, H., Jain, R., Khan, Y. A., Low, C. M. R., Perlin, K., Potapenko, A., Savy, P., Singh, S., Stecula, A., Thillaisundaram, A., Tong, C., Yakneen, S., Zhong, E. D., Zielinski, M., Zidek, A., Bapst, V., Kohli, P., Jaderberg, M., Hassabis, D., and Jumper, J. M. (2024) Accurate structure prediction of biomolecular interactions with AlphaFold 3. *Nature* **630**, 493-500
9. Hanahan, D. (1983) Studies on transformation of *Escherichia coli* with plasmids. *J Mol Biol* **166**, 557-580
10. Studier, F. W., and Moffatt, B. A. (1986) Use of bacteriophage T7 RNA polymerase to direct selective high-level expression of cloned genes. *J. Mol. Biol.* **189**, 113-130
11. Wood, W. B. (1966) Host specificity of DNA produced by *Escherichia coli*: bacterial mutations affecting the restriction and modification of DNA. *J Mol Biol* **16**, 118-133
12. Sander, P., Meier, A., and Boettger, E. C. (1995) *rpsL*+: a dominant selectable marker for gene replacement in mycobacteria. *Mol. Microbiol.* **16**, 991-1000
13. Flores, A. R., Parsons, L. M., and Pavelka, M. S., Jr. (2005) Genetic analysis of the beta-lactamases of *Mycobacterium tuberculosis* and *Mycobacterium smegmatis* and susceptibility to beta-lactam antibiotics. *Microbiology* **151**, 521-532
14. Kaps, I., Ehrt, S., Seeber, S., Schnappinger, D., Martin, C., Riley, L. W., and Niederweis, M. (2001) Energy transfer between fluorescent proteins using a co-expression system in *Mycobacterium smegmatis*. *Gene* **278**, 115-124
15. Stephan, J., Bender, J., Wolschendorf, F., Hoffmann, C., Roth, E., Mailänder, C., Engelhardt, H., and Niederweis, M. (2005) The growth rate of *Mycobacterium smegmatis* depends on sufficient porin-mediated influx of nutrients. *Mol. Microbiol.* **58**, 714-730
